# Perinatal mortality in eastern Africa: A systematic review and meta‐analysis

**DOI:** 10.1002/ijgo.70717

**Published:** 2026-01-23

**Authors:** Yohanis Alemeshet Asefa, Assefa Tola Gemeda, Hannah Blencowe, Nega Assefa, Lars Åke Persson, Anna C. Seale

**Affiliations:** ^1^ Department of Disease Control London School of Hygiene and Tropical Medicine London UK; ^2^ School of Environmental Health, College of Health and Medical Science Haramaya University Harar Ethiopia; ^3^ School of Public Health, College of Health and Medical Science Haramaya University Harar Ethiopia; ^4^ Department of Infectious Disease Epidemiology and International Health London School of Hygiene and Tropical Medicine London UK; ^5^ School of Nursing and Midwifery, College of Health and Medical Science Haramaya University Harar Ethiopia; ^6^ Health System and Reproductive Health Research Directorate Ethiopian Public Health Institute Addis Ababa Ethiopia; ^7^ Faculty of Epidemiology and Population Health Warwick Medical School, University of Warwick Coventry UK

**Keywords:** early neonatal mortality, East Africa, perinatal mortality, stillbirth, systematic review

## Abstract

**Background:**

Every day, over 5000 stillbirths and more than 6000 newborn deaths are estimated to occur worldwide, the majority in sub‐Saharan Africa and South Asia. Estimates, however, rely on data that might underestimate these deaths. Further, outside major categories such as preterm birth, infection, and complications at delivery, little is known about the causes of death for newborns and even less for stillbirths.

**Objectives:**

This systematic review and meta‐analysis aimed to synthesize evidence on the incidence, causes, and risk factors for perinatal mortality in East Africa to inform public health policy.

**Method:**

We searched major databases: Medline, Web of Science, EMBASE, Global Health, SCOPUS, Cochrane Library, CINAHL, HINARI, African Index Medicus, African Journals Online (AJOL), DHS website, and the World Health Organization African Regional Office (AFRO) Library. The search was conducted without imposing any language restrictions. Studies published 2010–2022 reporting perinatal mortality (incidence/causes/risk factors) in East African countries were included. We included all observational studies (cross‐sectional, case–control, prospective cohort, and retrospective studies) and community‐based trials. We performed meta‐analyses with random effects to estimate pooled perinatal mortality rates for the population and health facility settings. We investigated and reduced heterogeneity where appropriate. We summarized causes of death descriptively and synthesized risk factors narratively.

**Results:**

We included 99 out of 22 889 studies. The pooled population‐based perinatal mortality rate was 33.1/1000 births (95% confidence interval [CI]: 29.3–37.1, *I* ^2^ = 96.9%), and for health facility settings 67.1/1000 (95% CI: 47.5, 89.7, *I* ^2^ = 98.9%). The major reported causes of perinatal deaths were infections and intrapartum‐related events. However, our understanding of the specific causes of death was limited by the lack of detailed information necessary for diagnosing specific causes. Risk factors for perinatal mortality were demographic (low socioeconomic status), related to care (lack of antenatal care, obstetric complications), and maternal comorbidities. Protective factors included women's empowerment and supporting access to care through maternity waiting homes.

**Conclusion:**

Perinatal mortality remains high in East Africa. Many deaths were preventable through addressing modifiable risk factors and strengthening health systems to provide quality antenatal and intrapartum care. Consistent use of standardized cause‐of‐death classification and improved data quality are needed to enhance the understanding of specific causes of death and target interventions.

## 1 INTRODUCTION

Perinatal mortality, defined as the death of a fetus from 28 weeks' gestation and a neonate up to 7 days after birth,^1^ remains a significant global health challenge. Daily, over 5000 stillbirths and more than 6000 newborn deaths occur worldwide.^2^, ^3^ These deaths disproportionately occur in sub‐Saharan Africa and South Asia.^4^, ^5^ However, South Asia has seen a decline in stillbirths and neonatal mortality, while sub‐Saharan Africa has made the least progress, with the highest stillbirth and neonatal mortality rates globally.^2^, ^6^ Most sub‐Saharan countries are unlikely to achieve the Every Newborn Action Plan (ENAP) goal, which sets the global target of reducing the neonatal mortality rate to 12 or fewer per 1000 live births and stillbirths to 12 or fewer per 1000 total births by 2030.^2^ Regional data to guide targeted interventions are required to support progress toward these goals.^7^, ^8^

East Africa has the most fragile economy in sub‐Saharan Africa, with an average GDP per capita of $2983, much lower than the average of the emerging markets and developing economies.^9^, ^10^ Further, East Africa has the lowest maternal continuum of care (17.5%), which includes the utilization of maternal healthcare services, including four or more antenatal care visits, skilled birth attendance, and postnatal care, compared to West and South Africa.^11^

Data collection methodologies limit accurate estimation of perinatal mortality rates in East Africa. Civil Registration and Vital Statistics (CRVS) systems are weak, and estimates of perinatal mortality mainly come from specific studies or surveys, such as the Demographic and Health Survey (DHS).^12^, ^13^ A meta‐analysis of DHS data from 2010 to 2016 indicated that East Africa had the highest perinatal mortality rate, with 34.1 deaths per 1000 births.^14^ However, estimates based on these data alone are prone to underestimation through recall bias because data are cross‐sectionally collected with long recall periods (3–5 years).^15^ An estimate from the Global Burden of Disease, covering 1990 to 2021, indicated that the stillbirth rate was highest in East Africa, at 32/1000 births, with stillbirth defined as the death of a fetus from 20 weeks of gestation. Although this modeled estimate used a range of data points, its precision might be limited by poor data quality, particularly from low‐income settings.^16^

Previous reviews on perinatal mortality in sub‐Saharan Africa have been limited in scope by focusing on either stillbirth or neonatal mortality, making it difficult to define the actual burden because perinatal deaths are frequently misclassified between stillbirth and very early neonatal deaths.^17^ Hence, this study focused on perinatal mortality, which is a combination of stillbirths and early neonatal mortality (deaths of livebirths in the first week of life).^18^, ^19^ Further, previous reviews were mainly based on demographic and health survey data, and only English‐language studies were included.^14^, ^20^, ^21^, ^22^ In addition to the major causes of neonatal deaths, such as preterm birth, infections, and complications at delivery,^23^ little is known about the specific causes of newborn deaths and even less for stillbirths.

We undertook a systematic review and meta‐analysis to provide an up‐to‐date estimate of perinatal mortality. Moreover, synthesizing the causes and risk factors is important to guide intervention strategies. Therefore, this study aims to synthesize published and unpublished evidence on the incidence, causes, and risk factors for perinatal mortality in Eastern Africa to inform public health policy.

## 2 METHODS

The protocol for this systematic review and meta‐analysis was published in alignment with the Preferred Reporting Items for Systematic Reviews and Meta‐Analyses (PRISMA) guidelines^24^ and is registered with PROSPERO (number CRD42021291719). We report our findings according to the PRISMA guideline (Appendix S1).^25^

### 2.1 Eligibility criteria

This study included both published and unpublished observational studies (cross‐sectional, case–control, prospective cohort, and retrospective studies) and community‐based trials, which reported the incidence, causes, or risk factors of at least one of the outcomes (stillbirths, early neonatal mortality, or perinatal mortality). Because there are a variety of different definitions for stillbirths, we included studies that define stillbirth starting from ≥500 g/≥22 weeks of gestation and newborn deaths within the first week after birth (0–6 days). We included papers published in any language between 2012 and 2022 in East Africa. The list of countries included in this study can be found in Appendix S2.

We excluded review articles and studies on specific sub‐group populations (e.g., high‐risk mothers or studies on pregnancies with specific complications). Studies were excluded if data extraction failed after appropriate attempts to secure the complete text, including efforts to contact the corresponding author when needed. We also excluded studies that were limited in methodology (e.g., with inappropriate statistical analysis or methods used to control confounders) and studies that did not define stillbirth.

### 2.2 Information sources

We searched databases to identify eligible studies in Medline, Web of Science, EMBASE, Global Health, SCOPUS, Cochrane Library, CINAHL, HINARI, African Index Medicus, African Journals Online (AJOL), DHS website, and the World Health Organization (WHO) African Regional Office (AFRO) Library. In addition, a manual search was performed to retrieve unpublished studies and gray literature via Google Scholar, Google, and institutional repositories of higher education institutions.

### 2.3 Search strategy

We searched using the following key terms to identify papers on the burden of perinatal mortality, its cause, and determinants in Eastern Africa: “perinatal mortality,” “perinatal death(s),” stillbirth(s), stillborn(s), “fetal death(s),” “fetal demise,” “fetal mortality,” “neonatal death(s),” “infant mortality,” and “East Africa.” These search terms were first developed for the Medline database, reviewed, and transcribed for other databases (Appendix S3). We also identified studies cited by others (descendent search strategy).

### 2.4 Selection process and data collection process

After deduplication using Endnote X20, two reviewers (YAA and ATG) independently screened titles, abstracts, and full text using Covidence software.^26^

Information was extracted regarding the title, author, publication year, study design, study setting, sample size, study participants, study period, and outcome of interest (definition of outcomes). In extracting data on perinatal mortality rates, we also separately gathered the rates of stillbirths and early neonatal deaths. Additionally, we extracted the time of death for stillbirths (antepartum or intrapartum). Data from multi‐country studies were presented by country where possible. Further, we examined the identification of causes and risk factors in each study, and the classification system was documented, if applicable.

### 2.5 Quality assessment and risk of bias assessment

The methodological quality of studies was assessed using the Joanna Briggs Institute (JBI) tool.^27^ Studies were classified as high (above 80%), moderate (60–80%), and low quality (under 60%). For prevalence studies, the risk of bias was assessed using the tool developed by Hoy et al.^28^ Studies that had positive responses to nine or more of the 10 questions were classified as having a “low risk of bias.” Responses indicating “yes” for seven to eight out of 10 questions were classified as “moderate risk,” and responses indicating “yes” for six or fewer out of 10 questions were classified as “high risk.” Further, the Risk of Bias in Non‐Randomized Studies—of Exposures (ROBINS‐E) framework was used to assess the risk of bias for risk factor studies.^29^ Two authors (YAA and ATG) conducted all assessments separately, and when discrepancies arose, a third author (LAP or ACS) facilitated discussions to reach a consensus.

### 2.6 Data synthesis and analysis

We conducted meta‐analyses with random effects to account for true variation between studies. We estimated the pooled perinatal mortality rate among studies that reported stillbirths (≥28 weeks) and early neonatal deaths (death within the first week), with a total number of births as the denominator. We also separately estimated stillbirth and early neonatal death rates using studies reporting these results with their appropriate denominator, total births, and total live births, respectively.

In these meta‐analyses, we quantified the proportion of overall variation attributable to inter‐study heterogeneity using the *I* ^2^ statistic.^30^ The *I* ^2^ values of 25%, 50%, and 75% were classified as low, moderate, and high heterogeneity, respectively. Because *I* ^2^ is not an absolute measure of heterogeneity,^31^ the prediction interval, providing a more meaningful measure, was calculated. Sub‐group analysis, meta‐regression, and sensitivity analyses were performed to assess sources of heterogeneity among studies. Pre‐determined subgroup analyses included study setting (population‐based vs. healthcare facility‐based), scope, economy, study period (before/after implementation of ENAP [2014]), study design, and risk of bias. We performed meta‐regression analysis with random effects to examine the origins of heterogeneity among population‐based studies. Factors with a *P*‐value ≤0.20 in univariable analysis were included in the multivariable analysis. Publication bias was assessed using a visual examination of funnel plots, the Egger test, and the trim‐and‐fill technique.^32^, ^33^ All statistical analyses were conducted using Stata (version 18).^34^

We summarized causes of death descriptively and synthesized risk factors narratively and by forest plots. We also updated the conceptual framework according to our findings.^24^

## 3 RESULTS

### 3.1 Study selection

**FIGURE 1:**
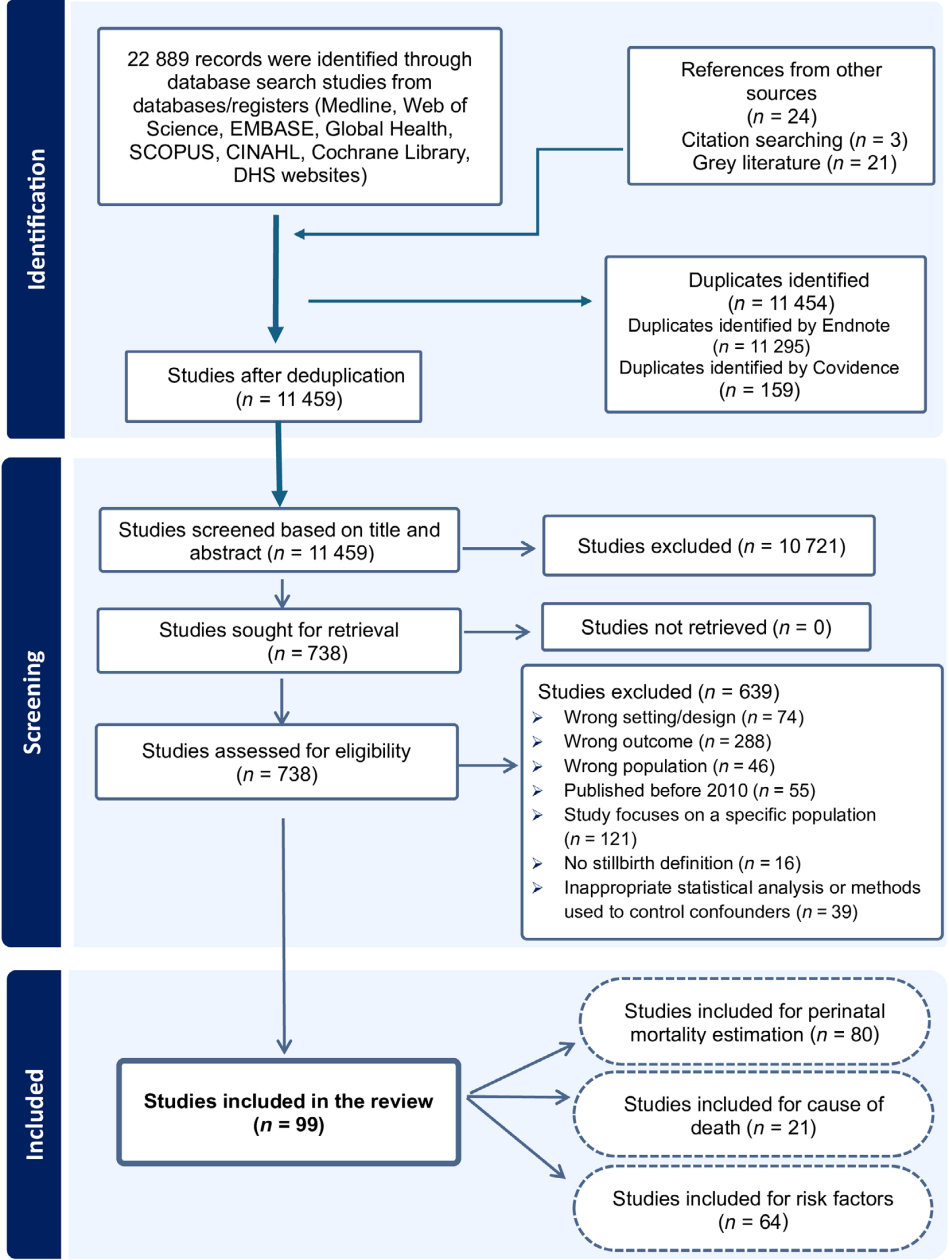
Studies included in the review of perinatal mortality in East Africa, summarizing the screening process (study could be included in more than one analysis).

We identified 22 913 articles (22 889 from database searches and 24 other sources). Ninety‐nine studies met the inclusion criteria after removing 11 454 duplicates and screening 11 459. Studies were published in English, except for two in Portuguese.^35^, ^36^ One study in French was excluded because of methodological limitations (Figure 1).^37^

### 3.2 Study characteristics

**FIGURE 2:**
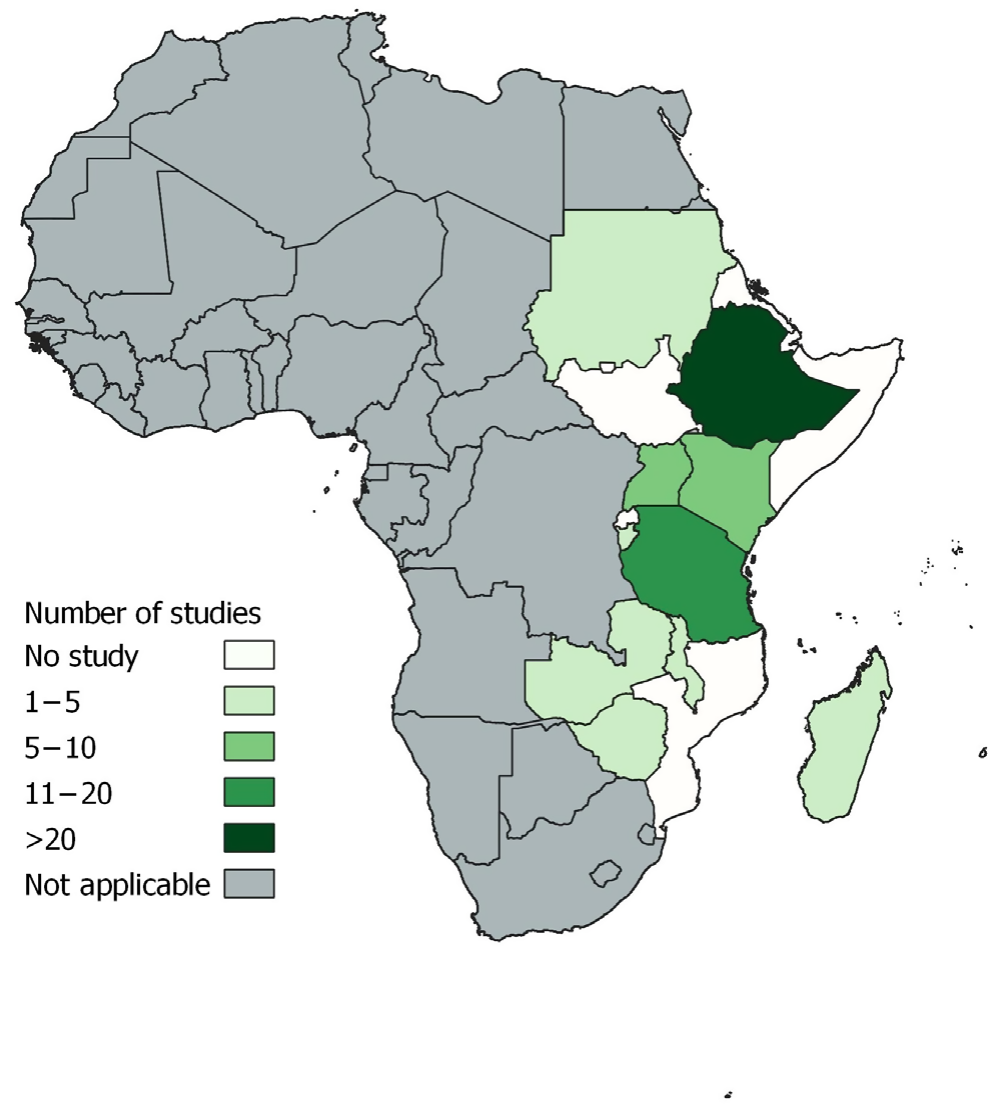
Map of Africa showing the number of studies within East African countries included in this systematic review and meta‐analysis of perinatal mortality within East African countries.

Of the 99 included studies, more than half used a cross‐sectional design,^35^, ^36^, ^38^, ^39^, ^40^, ^41^, ^42^, ^43^, ^44^, ^45^, ^46^, ^47^, ^48^, ^49^, ^50^, ^51^, ^52^, ^53^, ^54^, ^55^, ^56^, ^57^, ^58^, ^59^, ^60^, ^61^, ^62^, ^63^, ^64^, ^65^, ^66^, ^67^, ^68^, ^69^, ^70^, ^71^, ^72^, ^73^, ^74^, ^75^, ^76^, ^77^, ^78^, ^79^, ^80^, ^81^, ^82^, ^83^, ^84^, ^85^, ^86^, ^87^ while others employed cohort,^88^, ^89^, ^90^, ^91^, ^92^, ^93^, ^94^, ^95^, ^96^, ^97^, ^98^, ^99^, ^100^, ^101^, ^102^, ^103^, ^104^, ^105^, ^106^, ^107^, ^108^, ^109^, ^110^, ^111^, ^112^, ^113^, ^114^, ^115^ case–control,^116^, ^117^, ^118^, ^119^, ^120^, ^121^, ^122^, ^123^, ^124^, ^125^, ^126^, ^127^, ^128^, ^129^, ^130^, ^131^ case series,^132^, ^133^ and community‐based trial designs.^134^ More than half the studies were conducted in Ethiopia (*n* = 55) followed by Tanzania (*n* = 16) and Uganda (*n* = 9). The remaining studies were conducted in Kenya, Malawi, Zambia, Rwanda, Zimbabwe, Burundi, Madagascar, and Sudan (Figure 2). Fifty‐four studies were facility‐based, while the remaining 45 were population‐based (Appendix S4).

### 3.3 Quality assessment and risk of bias

All studies were graded moderate to high quality on JBI criteria (Appendix S5, Table S5.1–S5.5).

Among the 34 studies (included for the pooled perinatal mortality rate), 17 (50%) had low risk of bias, 13 (38.2%) a moderate risk, and four (11.8%) a high risk of bias (Appendix S5, Table S5.6).

ROBINS‐E assessment of risk factor studies revealed concern in confounding (36/64 studies) and selection bias (37 studies). A moderate risk of bias was identified in three domains: bias arising from the measurement of exposure (10 studies), outcome measurement (21 studies), and missing data (eight studies), with each study showing either some concerns or a high risk of bias. However, nearly all studies demonstrated a low risk of bias for post‐exposure interventions and in selective reporting (Appendix S5, Figure S5.1 and Figure S5.2).

### 3.4 Pooled estimates of perinatal mortality

**FIGURE 3:**
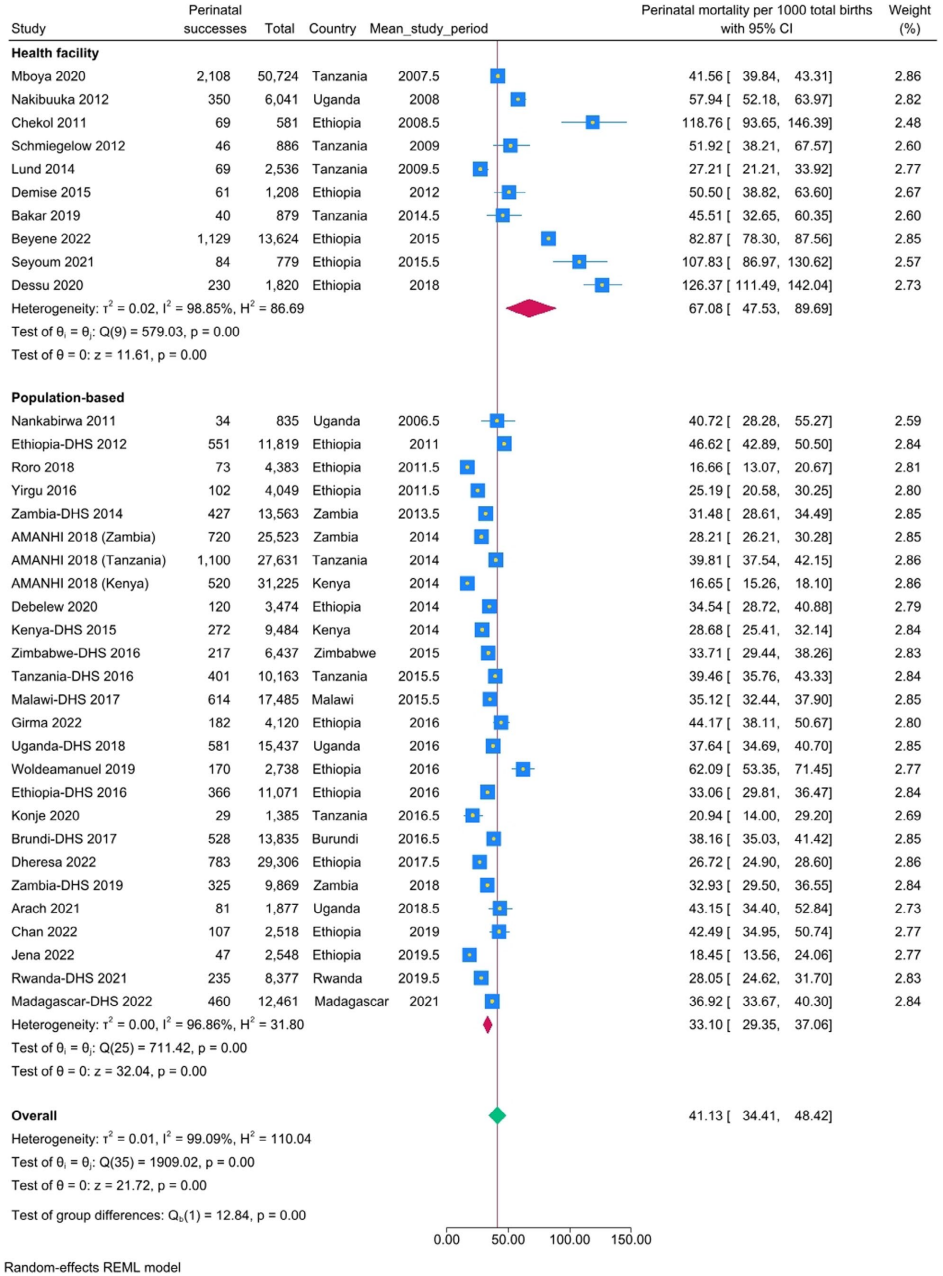
Meta‐analysis showing pooled perinatal mortality rates in population‐based settings and health facilities in East Africa (2010–2022).

The pooled perinatal mortality rate in population‐based studies (*n* = 26) was 33.1 per 1000 births (95% confidence interval [CI]: 29.4, 37.1). For facility‐based studies (*n* = 10) it was 67.1 per 1000 births (95% CI: 47.5, 89.7) (Figure 3).

The population‐based stillbirth and early neonatal mortality rates in East Africa were 17.2 per 1000 births (95% CI: 13.9, 20.1) and 19.0 per 1000 live births (95% CI: 16.8, 21.3), respectively. The stillbirth rate to early neonatal mortality rate ratio was 0.9. The facility‐based rates of stillbirth and early neonatal mortality were 49.1 per 1000 births (95% CI: 39.4, 59.8) and 44.3 per 1000 live births (95% CI: 33.1, 57.0), respectively (Appendix S6, Figure S6.1 and Figure S6.2).

### 3.5 Timing of stillbirths in East Africa

Of the 54 studies that reported stillbirth data (≥28 weeks gestation), only 14 studies, encompassing 4795 stillbirths, included information on the timing of stillbirths. For these, 2544 (53.1%) were categorized as antepartum (macerated) stillbirths, whereas 2251 (46.9%) occurred during the intrapartum period (fresh stillbirths).

### 3.6 Heterogeneity

Heterogeneity was high in estimates using both population‐based (*I* ^2^ = 96.9%, Galbraith plot Appendix S7) and facility‐based studies (*I* ^2^ = 98.9%). The prediction interval was 16 to 56 deaths per 1000 total births (for population‐based studies) and 10 to 169 deaths per 1000 total births for facility‐based studies.

### 3.7 Sub‐group analysis

In population‐based studies, higher perinatal mortality rates were seen among countrywide studies, studies conducted after the implementation of ENAP (after 2014), cross‐sectional studies, and studies from lower‐income countries. Sub‐groups with high perinatal mortality rates among health facility‐based studies included studies conducted after the implementation of ENAP (after 2014), cross‐sectional studies, studies from low‐income countries, studies with a high risk of bias, and studies from referral health facilities. Heterogeneity persisted among all sub‐groups, indicating that these variables alone did not fully explain the heterogeneity in perinatal mortality rates across studies (detailed results in Appendix S8, Table S8.1–S8.2).

### 3.8 Origins of heterogeneity

In the final multivariable model of meta‐regression, study design, study period (before/after implementation of ENAP [2014]), risk of bias, and place of residence were potential moderators. The meta‐regression model accounted for 19.5% of the variation across studies, with residence (studies conducted in urban and rural areas) (β = 0.15, *P* = 0.031) identified as the only significant moderator (Appendix S9, Table S9.1 and Table S9.2).

### 3.9 Sensitivity analysis

We found that omitting any individual study by leave‐one‐out sensitivity analysis among the population‐based and health facility‐based studies did not significantly affect the overall pooled estimate (Appendix S10).

### 3.10 Publication bias

There was asymmetry in the funnel of population‐based perinatal mortality rates, indicating potential bias (Appendix S11, Figure S11.1); and the trim and fill approach imputed three studies (Appendix S11, Figure S11.2). An Egger's test with no moderator variable revealed no significant small‐study effect (*P* = 0.6). However, when controlling for moderators (study period and risk of bias), there was substantial evidence of small‐study effects (*P* = 0.0025).

### 3.11 Causes of perinatal mortality

Twenty‐one studies from 10 countries reported causes of perinatal death, comprising 32 489 perinatal deaths (characteristics in Appendix S12). Most studies did not use a classification system. Although eight studies were conducted after the introduction of the International Classification of Diseases for Perinatal Mortality (ICD‐PM), only four used this classification system.^55^, ^79^, ^90^, ^132^

**FIGURE 4:**
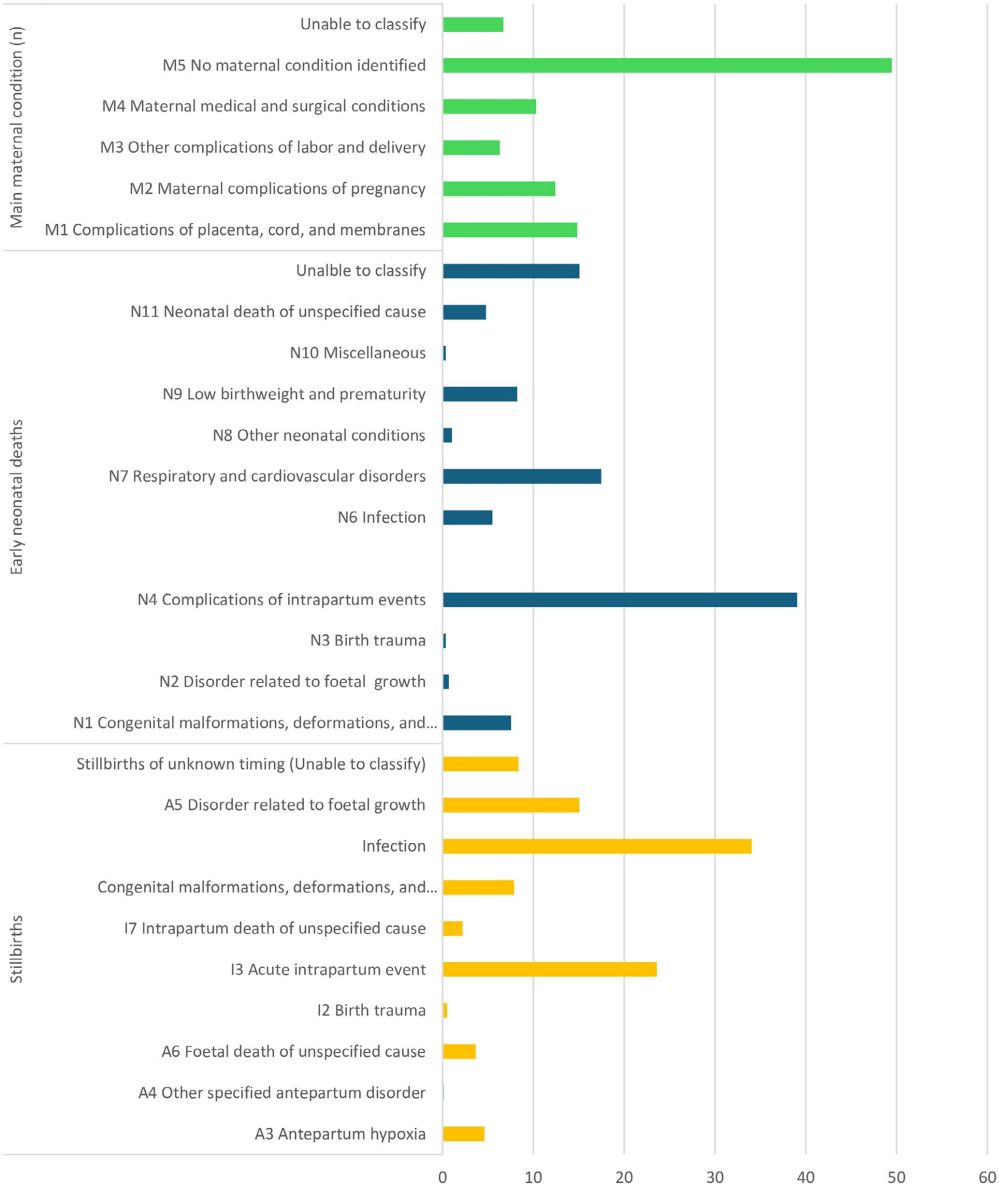
Reported causes of perinatal deaths according to International Classification of Diseases for Perinatal Mortality (ICD‐PM) classification system in East Africa (2010–2022).

**FIGURE 5:**
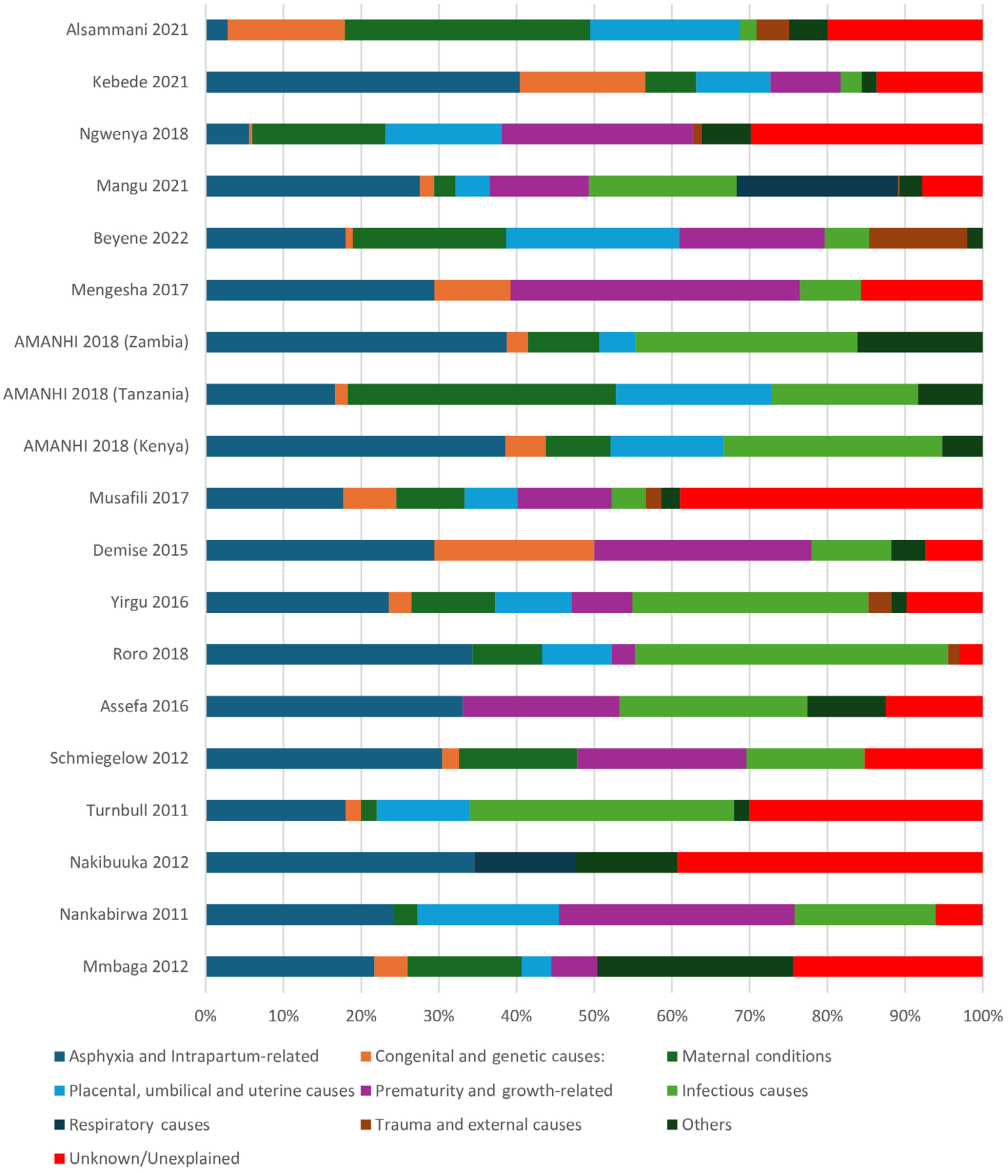
Reported causes of perinatal deaths among studies that used classification systems other than International Classification of Diseases for Perinatal Mortality (ICD‐PM) (2010–2022).

All four studies using ICD‐PM had health professionals assigned cause of death, with three studies^55^, ^79^, ^132^ using health facility records and one population‐based study^90^ using verbal autopsy.^55^, ^79^, ^132^ The result was summarized from three studies^55^, ^79^, ^90^ as one of the studies,^132^ combines results with findings from outside East Africa (Sierra Leone). The associated maternal conditions were not reported in one study.^90^ The most commonly reported causes of stillbirth were infections, followed by acute intrapartum events and disorders related to fetal growth.^79^, ^90^, ^132^ Complications of intrapartum events were the leading cause of early neonatal mortality, followed by respiratory and cardiovascular disorders, low birth weight, and prematurity. For associated maternal conditions, complications of the placenta, cord, and membranes were commonest, followed by maternal complications of pregnancy and maternal medical and surgical conditions (Figure 4). The remaining 17 studies used a variety of classification systems.^41^, ^45^, ^47^, ^66^, ^69^, ^71^, ^72^, ^80^, ^91^, ^93^, ^111^, ^113^, ^114^, ^124^, ^129^, ^131^, ^133^ Among these studies, asphyxia and intrapartum‐related causes accounted for the highest percentage of perinatal deaths, followed by infections, prematurity, growth‐related conditions, and unexplained causes (Figure 5).

### 3.12 Risk factors of perinatal mortality

Our systematic review identified a range of risk factors associated with perinatal mortality, and we organized these according to the conceptual model developed in the published protocol (Appendix S13).^24^

### 3.13 Socio‐demographic, economic, and lifestyle factors

Education and income level showed mixed associations with perinatal mortality. In several studies, lower maternal education was linked to increased risk of having perinatal mortality,^40^, ^44^, ^57^, ^61^, ^78^, ^92^, ^110^, ^124^, ^125^, ^130^ while others found no association.^42^, ^46^, ^63^, ^83^, ^84^, ^90^, ^100^, ^114^, ^116^, ^118^, ^129^, ^131^ Similarly, lower income was associated with increased perinatal mortality in some studies (aOR 1.7–3.3),^61^, ^83^, ^84^ while others found no association.^53^, ^57^, ^78^, ^90^, ^100^, ^118^, ^128^, ^131^

Rural residence was associated with higher risk (aOR 1.2–4.8),^38^, ^44^, ^57^, ^63^, ^78^, ^83^, ^84^, ^100^, ^110^, ^128^ although several found no association^46^, ^48^, ^53^, ^68^, ^74^, ^116^, ^118^, ^119^, ^123^ or even protective effects.^113^ A limited number of studies on water access and sanitation suggested links between poor sanitation infrastructure and increased risk (aOR 1.4–1.6).^40^, ^83^

Family size showed contradictory results, with one study reporting a higher risk for larger families,^38^ while others found protective effects (OR 0.06–0.2).^53^, ^88^, ^131^ Maternal alcohol consumption during pregnancy was consistently associated with increased risk of perinatal mortality (aOR 1.4–3.0),^88^, ^96^, ^126^ except in one study,^110^ and smokers had a higher risk compared to non‐smokers.^40^

Marital status revealed no association,^42^, ^57^, ^124^, ^129^ and occupation showed a mixed result in which one study showed that being a farmer increased the risk of having perinatal mortality,^38^ while others showed no association.^44^, ^46^, ^100^, ^129^, ^131^

Women's participation in healthcare decisions (women empowerment)^53^ and female‐headed households^84^ were associated with lower risk. Intimate partner violence showed an increased risk (aOR 3.3),^52^ while unintended pregnancies had no associations.^63^ The association with exposure to mass media was inconsistent across studies.^53^, ^78^ (Appendix S14, Figure S14.1).

### 3.14 Obstetric risk factors

Advanced maternal age (≥30–35 years) generally increases the risk of perinatal mortality (aOR 1.2–7.6).^40^, ^43^, ^44^, ^57^, ^61^, ^84^, ^90^, ^92^, ^96^, ^98^, ^110^, ^112^, ^128^, ^129^ The risk was particularly pronounced for women over 40 years.^42^, ^83^ However, some studies showed no association.^45^, ^46^, ^70^, ^100^, ^114^, ^118^, ^123^, ^125^, ^131^

Some studies found higher perinatal mortality risk for nulliparous women or primiparity,^90^, ^100^, ^118^, ^131^ while others reported increased risk for grand multiparity.^53^, ^60^, ^83^, ^97^, ^118^ Many studies showed no association for various parity categories.^48^, ^49^, ^78^, ^88^, ^90^, ^114^, ^123^, ^124^, ^129^ Conversely, compared to having at least one child, nulliparous or primiparous women had a reduced risk of having perinatal deaths in two studies.^44^, ^74^

Lack of antenatal care (ANC) or inadequate ANC (fewer than four ANC visits) were associated with an increased risk of perinatal deaths,^38^, ^44^, ^48^, ^49^, ^53^, ^70^, ^74^, ^78^, ^84^, ^96^, ^100^, ^110^, ^114^, ^116^, ^118^, ^119^, ^120^, ^122^, ^123^, ^125^ while some studies found no significant association.^42^, ^43^, ^46^, ^63^, ^68^, ^90^, ^124^, ^129^ Gwako et al.^122^ found that specific quality indicators of ANC (i.e., having a measurement for hemoglobin, blood group, HIV, VDRL, and weight) were associated with lower stillbirth risk (aOR: 0.2–0.4).

Multiple gestations consistently showed higher perinatal mortality risk compared to singleton pregnancies, with odds ratios ranging from 2.6 to 5.6.^60^, ^83^, ^116^, ^118^, ^129^ Short interpregnancy (birth) intervals (<24 months) were associated with increased stillbirth risk in several studies.^53^, ^60^, ^61^, ^78^, ^83^, ^84^, ^104^, ^105^, ^109^, ^120^, ^127^ Some studies found no observed effect of multiple gestation^124^, ^131^ and interpregnancy (inter‐birth) interval on perinatal mortality.^102^, ^129^, ^131^

Modes of delivery had inconsistent results. In some studies, the risk of perinatal deaths was significantly increased with caesarean section,^48^, ^78^, ^101^, ^116^ while others found protective effects^44^, ^45^, ^96^, ^110^, ^119^, ^127^ and many studies have shown no association.^49^, ^74^, ^124^, ^131^

Several studies reported significantly increased perinatal deaths with maternal anemia,^44^, ^48^, ^96^, ^120^, ^123^ and the adjusted odds ratios increased significantly for hemoglobin levels <8 gm/dL.^114^ However, many studies found no association between anemia and perinatal death.^63^, ^68^, ^70^, ^88^, ^119^, ^125^ Studies found no association between maternal obesity and perinatal mortality.^44^, ^90^ One study reported an increased risk of stillbirth among mothers with undernutrition (MUAC <21 cm),^63^ while another showed no association.^114^

Previous adverse pregnancy outcomes (pregnancy termination, preterm birth, stillbirth, or neonatal mortality) generally increased the perinatal mortality risk in subsequent pregnancies,^38^, ^49^, ^53^, ^57^, ^60^, ^70^, ^84^, ^118^, ^123^, ^124^, ^125^, ^126^, ^129^, ^130^, ^131^ while few studies found no association.^74^, ^119^

One study on sexually transmitted infections showed increased stillbirth risk,^130^ and most studies showed that HIV infection had no association with perinatal mortality,^46^, ^83^, ^96^, ^116^ except among HIV‐infected women not on Antiretroviral Therapy (ART), which increased the risk.^81^ (Refer to Appendix S14, Figure S14.2).

### 3.15 Obstetric complications

Hypertensive disorders of pregnancy showed an increased risk of perinatal mortality with aOR ranging from 1.1 to 9.5, ^38^, ^68^, ^70^, ^94^, ^96^, ^103^, ^110^, ^116^, ^121^, ^125^, ^127^ while a few studies found no association.^115^, ^119^, ^123^, ^124^ One study found that prepartum onset of pregnancy‐induced hypertension carried a higher risk than postpartum onset (aOR: 4.0; 2.0, 6.0).^48^ For gestational diabetes mellitus, one study showed increased risk,^119^ but this was not consistently observed.^96^

Obstructed labor was associated with higher risk of perinatal mortality (aOR: 2.6–3.5),^68^, ^70^, ^119^ except for a study in Ethiopia.^127^ Prolonged labor (>18/24 h) substantially increased the risk of perinatal deaths in most studies (aOR: 3.2–6.5),^119^, ^121^, ^127^ with only one study finding no association.^116^ Malpresentation showed an increased risk of perinatal mortality.^96^, ^121^

Antepartum hemorrhage was strongly associated with increased perinatal mortality (aORs: 2.4–18.7).^38^, ^68^, ^70^, ^110^, ^119^, ^124^, ^126^ Premature rupture of membranes showed mixed results. While some studies reported increased risk (aOR: 1.7–4.0),^68^, ^126^, ^130^ others found no association^70^, ^116^, ^124^, ^127^ or even a protective effect.^96^, ^110^ (Refer to Appendix S14, Figure S14.3).

### 3.16 Fetal factors

Preterm birth^38^, ^43^, ^48^, ^68^, ^70^, ^88^, ^96^, ^110^, ^114^, ^116^, ^120^, ^129^, ^131^ and low birth weight^38^, ^48^, ^52^, ^56^, ^57^, ^63^, ^68^, ^96^, ^100^, ^110^, ^126^, ^127^ consistently emerged as a strong risk factor for perinatal mortality. However, some studies reported no associations.^63^, ^119^, ^121^, ^123^ Higher birth weight increased risk in some populations.^96^, ^100^ Small‐for‐gestational‐age status has also increased the risk of perinatal mortality (aOR: 3.5, 95% CI: 1.2–10.6).^114^

Congenital anomalies consistently showed associations with stillbirths, with aORs ranging from 3.1 to 9.7.^119^, ^121^, ^130^ Male sex demonstrates an increased risk of perinatal death, with aORs ranging from 1.1 to 5.5 in several studies.^60^, ^83^, ^110^, ^129^ However, other studies showed no association.^43^, ^57^, ^100^, ^110^, ^131^ (Refer to Appendix S14, Figure S14.4).

### 3.17 Health system factors

We found varied results for place of delivery, with some studies indicating an increased risk with home delivery (aOR ranging from 1.8 to 4.1),^44^, ^84^, ^113^ while others found no association^57^, ^106^ or reduced risk (aOR ranging from 0.07 to 0.7).^107^, ^129^, ^131^

Distance to health facilities and accessibility demonstrated inconsistent associations. Some studies found an increased risk with longer distances or perceived problems in access (aOR: 1.99, 95% CI: 1.2–3.2),^53^ while others showed no association^42^, ^84^, ^118^, ^129^ or even decreased risk (aOR: 0.55, 95% CI: 0.35–0.87).^62^ One study indicated that women coming directly from home had lower odds of perinatal mortality than those referred from health facilities,^123^ but another study showed no difference in perinatal mortality between self‐referred women and those referred from health facilities (aOR: 0.6, 95% CI: 0.33–1.05).^121^

Maternity waiting homes consistently showed a protective effect against perinatal mortality (aOR: 0.3–0.4),^89^, ^117^ indicating the ability to improve outcomes for high‐risk pregnancies. Failure to use partographs during childbirth was consistently linked with an increased risk of perinatal mortality (aOR ranging from 2.0 to 8.6),^43^, ^119^, ^120^, ^123^, ^126^, ^130^ with only one study finding no association.^127^ Labor induction or augmentation showed mixed results, with some studies reporting increased risk (aOR ranging from 3.0 to 3.8),^63^, ^74^, ^121^ while others identified no association.^48^, ^123^

Interventions such as mobile phone‐based programs were associated with reduced risk of perinatal mortality (aOR: 0.5, 95% CI: 0.27–0.93)^134^ However, a results‐based financing intervention was not associated with reduced risk of stillbirth.^64^ (Appendix S14, Figure S14.5).

## 4 DISCUSSION

Our systematic review and meta‐analysis showed a high population‐based perinatal mortality rate in East Africa (33.1/1000 births), and even higher incidence in facility‐based settings (67.1/1000). Cause of death data were limited by the standard of care available to support diagnoses, and most studies (17 out of 21) did not consistently classify cause of death using a standard classification system. Low socioeconomic status, obstetric risk factors, and obstetric complications were associated with increased perinatal mortality, while women's empowerment and use of maternity waiting homes were associated with reduced risk of perinatal mortality.

The pooled perinatal mortality rate in East Africa was high. However, the population‐based estimate is likely an underestimate, as there is underreporting, particularly for stillbirths, as shown by the ratio of the stillbirth rate to an early neonatal mortality rate of 0.9, which is less than the expected ratio of one; stillbirths are usually found to be at least equal to early neonatal deaths.^1^ Underreporting of stillbirths might arise due to recall bias or cultural influences.^135^ Further, data were rarely available from the most fragile areas, which typically have the highest death rates. No reports were available from Somalia, South Sudan, and Eritrea, and these data gaps might also have contributed to an underestimation. However, the estimates are the best available at present, with CRVS systems not well established. In the future, the availability of accurate data might improve as work is ongoing to enhance CRVS in similar settings, for example, in Mozambique.^136^

Our estimated perinatal mortality rate was much higher in facility‐based studies than population‐based studies. This might arise from referral of high‐risk women and/or women with complications seeking healthcare facilities. However, high mortality levels might reflect limited access to facilities and/or low quality of care. Within our estimates in these different settings, we found high heterogeneity. We tried to mitigate and understand the sources, but there remained unexplained variations in perinatal mortality rates across contexts. Strengthening the District Health Information Management System (DHIMS‐2) and triangulating information across data sources would better support accurate estimates of perinatal mortality.^137^, ^138^

In terms of the causes of perinatal deaths, studies used various classification systems, making comparisons difficult. Only four studies used the standard classification system (ICD‐PM), the WHO standard application of ICD‐10 for perinatal mortality, classifying intrapartum, antepartum, and neonatal deaths while identifying fetal and maternal causes. Among studies reporting causes of death, intrapartum complications and infections were most common. However, in these settings, clinical diagnoses are often unsupported by microbiological, advanced radiographic, or pathology approaches to fully inform the cause of death. There were also cases without clinical diagnoses, and while verbal autopsy (VA) can provide insights, it has limitations.^139^, ^140^ The VA is accurate for broad classifications, such as hemorrhage, but poor in defining specific causes, such as different infections. Both clinical diagnosis and verbal autopsy might underestimate specific causes of death, especially those associated with genetic anomalies, placental disorders, or infections. The reliance on these data sources, without post‐mortem autopsy, limits our understanding of the specific causes of death.^141^, ^142^ Complete post‐mortem autopsy is challenging in these settings, but there are specific programs, such as the Child Health and Mortality Prevention Surveillance (CHAMPS) program, aiming to determine specific causes of death using Minimal Invasive Tissue Sampling (MITS),^143^ combined with microbiological, pathological, and histological investigations. Going forward, these data could be used to calibrate VA, aiming to improve the information from this tool.

Risk factors for perinatal mortality identified in this review could be addressed. Socio‐demographic, economic, and lifestyle factors, such as low maternal education, low family wealth, and rural residency, were associated with perinatal mortality. These factors are interrelated and reflect social inequity. Further, not reported in previous reviews, women's empowerment was associated with a lower risk of perinatal mortality; empowered women are more likely to have timely health‐seeking behavior and make informed health decisions, thereby reducing perinatal mortality.^144^, ^145^

Access to intrapartum care and skilled birth attendance were associated with lower odds of perinatal mortality, highlighting the importance of the health system in decreasing perinatal mortality. However, the association between facility‐based delivery and perinatal mortality can vary with healthcare quality,^146^, ^147^, ^148^ health‐seeking behavior, and delays in referral.^149^, ^150^, ^151^ Most studies focus on receiving care, and overlook the quality of care. We found only one study assessing the benefit of quality antenatal care.^122^ At delivery, studies showed that no use of a partograph was associated with an increased risk of perinatal mortality, perhaps reflecting a lower standard of care.

## 5 CONCLUSION AND POLICY IMPLICATIONS

Perinatal death remains unacceptably high in East Africa. The leading reported causes of perinatal death were infections and intrapartum‐related events. However, understanding of specific causes of death is limited, with data mainly derived from routine clinical data and verbal autopsy, and comparability is limited without the use of the standardized classification system for causes of perinatal death. Women of higher socioeconomic groups and those with access to good antenatal and intrapartum care had a lower risk of perinatal death.

We recommend using standardization, prioritizing the use of ICD‐PM classification at all healthcare levels, and strengthening the existing Maternal and Perinatal Death Surveillance and Response. Further, fostering regional collaboration to support comprehensive and comparable data collection and analysis across East African countries is essential. Improving the accessibility and quality of maternal and newborn health services and addressing the broader social determinants of health, including women's empowerment and economic development, would likely reduce perinatal mortality.

## AUTHOR CONTRIBUTIONS

YAA conceived and designed the study, drafted the proposal, searched the databases, and conducted analysis in consultation with ACS, LAP, NA, and HB. YAA and ATG screened, selected, and extracted data and assessed the quality and risk of bias of studies. YAA prepared the manuscript, and ACS was the senior author who reviewed it critically. LAP, NA, and HB also reviewed and checked the manuscript for clarity. Finally, the manuscript was approved by all authors.

## FUNDING INFORMATION

The Gates Foundation (INV‐003659). The funder had no role in study design, data collection, analysis, interpretation, or preparation of the manuscript.

## CONFLICT OF INTEREST STATEMENT

The authors have no conflicts of interest.

## Supporting information


Appendix S1.

## Data Availability

All extracted datasets used for analysis, including the analysis code, will be accessible by contacting the corresponding author.
