# Supplementary material for: Perinatal mortality in eastern Africa: A systematic review and meta‐analysis
Source: Int J Gynaecol Obstet. 2026 Jan 23;173(3):1207–22. doi: 10.1002/ijgo.70717 (PMC13173618; doi:10.1002/ijgo.70717)

**Supplementary Appendices**

[**Supplementary Appendix 1: The PRISMA 2020 checklist** 1](#_Toc188307456)

[**Supplementary Appendix 2: Supplementary methods** 4](#_Toc188307457)

[**Supplementary Appendix 3: Search strategy in electronic databases** 5](#_Toc188307458)

[**Supplementary Appendix 4: Characteristics of Studies on perinatal mortality in East Africa.** 9](#_Toc188307459)

[**Supplementary Appendix 5: Quality assessment and risk of bias** 18](#_Toc188307460)

[**Supplementary Appendix 6: Pooled stillbirth rate and early neonatal mortality rate in East Africa.** 26](#_Toc188307461)

[**Supplementary Appendix 7: Galbraith plot studies included in population-based pooled perinatal mortality in East Africa.** 28](#_Toc188307462)

[**Supplementary Appendix 8: Sub-group analysis** 29](#_Toc188307463)

[**Supplementary Appendix 9: Meta-regression analysis** 31](#_Toc188307464)

[**Supplementary Appendix 10: Sensitivity analysis** 32](#_Toc188307465)

[**Supplementary Appendix 11: Publication bias** 33](#_Toc188307466)

[**Supplementary Appendix 12: Characteristics of studies on causes of perinatal mortality in East Africa.** 34](#_Toc188307467)

[**Supplementary Appendix 13: Updated conceptual framework for causes and risk factors of perinatal mortality.** 36](#_Toc188307468)

[**Supplementary Appendix 14: Forest plots of risk factors of perinatal mortality in East Africa.** 37](#_Toc188307469)

**Supplementary Appendix 1: The PRISMA 2020 checklist**

| **Section and Topic** | **Item #** | **Checklist item** | **Location where item is reported** |
| --- | --- | --- | --- |
| **TITLE** | | |  |
| Title | 1 | Identify the report as a systematic review. | 1 |
| **ABSTRACT** | | |  |
| Abstract | 2 | See the PRISMA 2020 for Abstracts checklist. | 2 |
| **INTRODUCTION** | | |  |
| Rationale | 3 | Describe the rationale for the review in the context of existing knowledge. | 3-4 |
| Objectives | 4 | Provide an explicit statement of the objective(s) or question(s) the review addresses. | 4 |
| **METHODS** | | |  |
| Eligibility criteria | 5 | Specify the inclusion and exclusion criteria for the review and how studies were grouped for the syntheses. | 4 & Supplementary Appendix 2 |
| Information sources | 6 | Specify all databases, registers, websites, organisations, reference lists and other sources searched or consulted to identify studies. Specify the date when each source was last searched or consulted. | 4 |
| Search strategy | 7 | Present the full search strategies for all databases, registers and websites, including any filters and limits used. | Supplementary Appendix 3 |
| Selection process | 8 | Specify the methods used to decide whether a study met the inclusion criteria of the review, including how many reviewers screened each record and each report retrieved, whether they worked independently, and if applicable, details of automation tools used in the process. | 5 |
| Data collection process | 9 | Specify the methods used to collect data from reports, including how many reviewers collected data from each report, whether they worked independently, any processes for obtaining or confirming data from study investigators, and if applicable, details of automation tools used in the process. | 5 |
| Data items | 10a | List and define all outcomes for which data were sought. Specify whether all results that were compatible with each outcome domain in each study were sought (e.g. for all measures, time points, analyses), and if not, the methods used to decide which results to collect. | 4-5 |
|  | 10b | List and define all other variables for which data were sought (e.g. participant and intervention characteristics, funding sources). Describe any assumptions made about any missing or unclear information. | 4-5 |
| Study risk of bias assessment | 11 | Specify the methods used to assess risk of bias in the included studies, including details of the tool(s) used, how many reviewers assessed each study and whether they worked independently, and if applicable, details of automation tools used in the process. | 5 |
| Effect measures | 12 | Specify for each outcome the effect measure(s) (e.g. risk ratio, mean difference) used in the synthesis or presentation of results. | 5-6 |
| Synthesis methods | 13a | Describe the processes used to decide which studies were eligible for each synthesis (e.g. tabulating the study intervention characteristics and comparing against the planned groups for each synthesis (item #5)). | 4-5 |
|  | 13b | Describe any methods required to prepare the data for presentation or synthesis, such as handling of missing summary statistics, or data conversions. | 5 |
|  | 13c | Describe any methods used to tabulate or visually display results of individual studies and syntheses. | 5 |
|  | 13d | Describe any methods used to synthesize results and provide a rationale for the choice(s). If meta-analysis was performed, describe the model(s), method(s) to identify the presence and extent of statistical heterogeneity, and software package(s) used. | 5-6 |
|  | 13e | Describe any methods used to explore possible causes of heterogeneity among study results (e.g. subgroup analysis, meta-regression). | 5-6 |
|  | 13f | Describe any sensitivity analyses conducted to assess robustness of the synthesized results. | 5-6 |
| Reporting bias assessment | 14 | Describe any methods used to assess risk of bias due to missing results in a synthesis (arising from reporting biases). | 5 |
| Certainty assessment | 15 | Describe any methods used to assess certainty (or confidence) in the body of evidence for an outcome. | 5 |
| **RESULTS** | | |  |
| Study selection | 16a | Describe the results of the search and selection process, from the number of records identified in the search to the number of studies included in the review, ideally using a flow diagram. | 6 |
|  | 16b | Cite studies that might appear to meet the inclusion criteria, but which were excluded, and explain why they were excluded. | 6 |
| Study characteristics | 17 | Cite each included study and present its characteristics. | Supplementary Appendix 4 |
| Risk of bias in studies | 18 | Present assessments of risk of bias for each included study. | 6 & Supplementary Appendix 5 |
| Results of individual studies | 19 | For all outcomes, present, for each study: (a) summary statistics for each group (where appropriate) and (b) an effect estimate and its precision (e.g. confidence/credible interval), ideally using structured tables or plots. | 7 |
| Results of syntheses | 20a | For each synthesis, briefly summarise the characteristics and risk of bias among contributing studies. | 6 |
|  | 20b | Present results of all statistical syntheses conducted. If meta-analysis was done, present for each the summary estimate and its precision (e.g. confidence/credible interval) and measures of statistical heterogeneity. If comparing groups, describe the direction of the effect. | 7-8 |
|  | 20c | Present results of all investigations of possible causes of heterogeneity among study results. | 7-8 |
|  | 20d | Present results of all sensitivity analyses conducted to assess the robustness of the synthesized results. | 8 |
| Reporting biases | 21 | Present assessments of risk of bias due to missing results (arising from reporting biases) for each synthesis assessed. | 8 |
| Certainty of evidence | 22 | Present assessments of certainty (or confidence) in the body of evidence for each outcome assessed. | 6 |
| **DISCUSSION** | | |  |
| Discussion | 23a | Provide a general interpretation of the results in the context of other evidence. | 12 |
|  | 23b | Discuss any limitations of the evidence included in the review. | 12-13 |
|  | 23c | Discuss any limitations of the review processes used. | 12-13 |
|  | 23d | Discuss implications of the results for practice, policy, and future research. | 14 |
| **OTHER INFORMATION** | | |  |
| Registration and protocol | 24a | Provide registration information for the review, including register name and registration number, or state that the review was not registered. | 4 |
|  | 24b | Indicate where the review protocol can be accessed, or state that a protocol was not prepared. | 4 |
|  | 24c | Describe and explain any amendments to information provided at registration or in the protocol. | n/a |
| Support | 25 | Describe sources of financial or non-financial support for the review, and the role of the funders or sponsors in the review. | n/a |
| Competing interests | 26 | Declare any competing interests of review authors. | 14 |
| Availability of data, code and other materials | 27 | Report which of the following are publicly available and where they can be found: template data collection forms; data extracted from included studies; data used for all analyses; analytic code; any other materials used in the review. | 14 |

*From:*  Page MJ, McKenzie JE, Bossuyt PM, Boutron I, Hoffmann TC, Mulrow CD, et al. The PRISMA 2020 statement: an updated guideline for reporting systematic reviews. BMJ 2021;372:n71. doi: 10.1136/bmj.n71

**Supplementary Appendix 2: Supplementary methods**

**List of East African countries included in this review**

We included studies conducted in East Africa, specifically Burundi, Comoros, Djibouti, Eritrea, Ethiopia, Kenya, Madagascar, Malawi, Mauritius, Mozambique, Rwanda, Seychelles, Somalia, Somaliland, South Sudan, Sudan, Tanzania, Uganda, Zambia, and Zimbabwe according to the United Nations’ definition.^1^

**Supplementary Appendix 3: Search strategy in electronic databases**

1. **Medline**

| **Component** | **Search terms** |
| --- | --- |
| 1 | Perinatal Mortality/ or stillbirth/ or fetal death/ or infant mortality/ |
| 2 | ((perinat* or f?etal or f?etus* or infant* or neonat*) adj5 (death* or mortalit* or demise)).mp. |
| 3 | (stillbirt* or stillborn* or adverse birth outcome* or pregnancy outcome* or perinatal outcome*).mp. |
| **4** | **1 Or 2 OR 3** |
| **5** | exp Africa, Eastern/ |
| 6 | (eastern Africa* or Burundi or Burundian or Comoros or Comorian or Djibouti or Djiboutian or Eritrea or Eritrean or Ethiopia or Ethiopian or Kenya or Kenyan or Madagascar or Malagasy or Madagascan or Malawi or Malawian or Mauritius or Mauritian or Mozambique or Mozambican or Rwanda or Rwandan or Seychelles or Seychellois or Somalia or Somalis or Somalian or Somaliland or South Sudan or Sudan or Sudanese or Tanzania or Tanzanian or Uganda or Ugandan or Zambia or Zambian or Zimbabwe or Zimbabwean).mp. |
| **7** | **5 OR 6** |
| **8** | **4 and 7** |
| **9** | **limit 8 to yr=”2010 - 2022”** |

1. **Web of Science**

| **Component** | **Search terms** |
| --- | --- |
| 1 | **TS=(“Perinatal Mortality” or stillbirth or “fetal death” or “infant mortality”)** |
| 2 | TS=(“Perinatal Mortality” or stillb* or “fetal death” or “infant mortality” AND (perinat* or f?etal* or f?etus* or infant* or neonat*) NEAR/5 (death* or mortalit* or demise)) |
| 3 | **TS=(stillbirt* or stillborn* or "adverse birth outcome*" or "pregnancy outcome*" or "perinatal outcome*")** |
| **4** | **1 Or 2 OR 3** |
| **5** | **TS=(Africa)** |
| 6 | **TS=(“**eastern Africa*” or Burundi or Burundian or Comoros or Comorian or Djibouti or Djiboutian or Eritrea or Eritrean or Ethiopia or Ethiopian or Kenya or Kenyan or Madagascar or Malagasy or Madagascan or Malawi or Malawian or Mauritius or Mauritian or Mozambique or Mozambican or Rwanda or Rwandan or Seychelles or Seychellois or Somalia or Somalis or Somalian or Somaliland or “South Sudan” or Sudan or Sudanese or Tanzania or Tanzanian or Uganda or Ugandan or Zambia or Zambian or Zimbabwe or Zimbabwean**)** |
| **7** | **5 OR 6** |
| **8** | **4 and 7** |
| **9** | **8 AND Publication date =(2010-01-01-2022-12-31)** |

1. **EMBASE**

| **Component** | **Search terms** |
| --- | --- |
| 1 | exp perinatal Mortality/ or stillbirth/ or fetal death/ or infant mortality/ |
| 2 | ((perinat* or f?etal or f?etus* or infant* or neonat*) adj5 (death* or mortalit* or demise)).mp. |
| 3 | (stillbirt* or stillborn* or adverse birth outcome* or pregnancy outcome* or perinatal outcome*).mp. |
| **4** | **1 OR 2 OR 3** |
| **5** | exp Africa, Eastern/ |
| 6 | (eastern Africa* or Burundi or Burundian or Comoros or Comorian or Djibouti or Djiboutian or Eritrea or Eritrean or Ethiopia or Ethiopian or Kenya or Kenyan or Madagascar or Malagasy or Madagascan or Malawi or Malawian or Mauritius or Mauritian or Mozambique or Mozambican or Rwanda or Rwandan or Seychelles or Seychellois or Somalia or Somalis or Somalian or Somaliland or South Sudan or Sudan or Sudanese or Tanzania or Tanzanian or Uganda or Ugandan or Zambia or Zambian or Zimbabwe or Zimbabwean).mp. |
| **7** | **5 OR 6** |
| **8** | **4 and 7** |
| **9** | **limit 8 to yr=”2010 - 2022”** |

1. **Global Health**

| **Component** | **Search terms** |
| --- | --- |
| 1 | Perinatal Mortality/ or stillbirth/ or fetal death/ or infant mortality/ |
| 2 | ((perinat* or f?etal or f?etus* or infant* or neonat*) adj5 (death* or mortalit* or demise)).mp. |
| 3 | (stillbirt* or stillborn* or adverse birth outcome* or pregnancy outcome* or perinatal outcome*).mp. |
| **4** | **1 Or 2 OR 3** |
| **5** | exp east Africa/ |
| 6 | (eastern Africa* or Burundi or Burundian or Comoros or Comorian or Djibouti or Djiboutian or Eritrea or Eritrean or Ethiopia or Ethiopian or Kenya or Kenyan or Madagascar or Malagasy or Madagascan or Malawi or Malawian or Mauritius or Mauritian or Mozambique or Mozambican or Rwanda or Rwandan or Seychelles or Seychellois or Somalia or Somalis or Somalian or Somaliland or South Sudan or Sudan or Sudanese or Tanzania or Tanzanian or Uganda or Ugandan or Zambia or Zambian or Zimbabwe or Zimbabwean).mp. |
| **7** | **5 OR 6** |
| **8** | **4 and 7** |
| **9** | **limit 8 to yr=”2010 - 2022”** |

1. **SCOPUS**

| **Component** | **Search terms** |
| --- | --- |
| 1 | TITLE-ABS-KEY({Perinatal Mortality} or stillbirth or {fetal death} or {infant mortality}) |
| 2 | TITLE-ABS-KEY({perinatal mortality} OR stillb* OR {fetal death} OR {infant mortality} OR {perinat* death} OR {perinat* mortali*} OR {fetal death*} OR {fetal mortal*} OR {fetal demise} OR {foetus* death*} OR {foetus* mortal*} OR {infant* death*} OR {infant mortal*} OR {neonat* death*} OR {neonat* mortal*}) |
| 3 | TITLE-ABS-KEY(stillbirt* or stillborn* or {adverse birth outcome*} or {pregnancy outcome*} or {perinatal outcome*}) |
| **4** | **1 Or 2 OR 3** |
| 5 | TITLE-ABS-KEY({eastern Africa*} or Burundi or Burundian or Comoros or Comorian or Djibouti or Djiboutian or Eritrea or Eritrean or Ethiopia or Ethiopian or Kenya or Kenyan or Madagascar or Malagasy or Madagascan or Malawi or Malawian or Mauritius or Mauritian or Mozambique or Mozambican or Rwanda or Rwandan or Seychelles or Seychellois or Somalia or Somalis or Somalian or Somaliland or {South Sudan} or Sudan or Sudanese or Tanzania or Tanzanian or Uganda or Ugandan or Zambia or Zambian or Zimbabwe or Zimbabwean) |
| **7** | **4 AND 5** |
| **8** | PUBYEAR > 2009 AND PUBYEAR < 2023 |
| **9** | 7 AND 8 |

1. **Cochrane Library**

| **Component** | **Search terms** |
| --- | --- |
| 1 | (perinatal NEXT (mortalit* or death* or outcome*)):ti,ab,kw |
| 2 | ((perinat* or f?etal or f?etus* or infant* or neonat*) NEXT (death* or mortalit* or demise)):ti,ab,kw |
| 3 | (stillbirt* or stillborn* or "adverse birth outcome*"):ti,ab,kw |
| 4 | #1 OR #2 OR #3 |
| 5 | (“eastern Africa*” or Burundi or Burundian or Comoros or Comorian or Djibouti or Djiboutian or Eritrea or Eritrean or Ethiopia or Ethiopian or Kenya or Kenyan or Madagascar or Malagasy or Madagascan or Malawi or Malawian or Mauritius or Mauritian or Mozambique or Mozambican or Rwanda or Rwandan or Seychelles or Seychellois or Somalia or Somalis or Somalian or Somaliland or “South Sudan” or Sudan or Sudanese or Tanzania or Tanzanian or Uganda or Ugandan or Zambia or Zambian or Zimbabwe or Zimbabwean):ti,ab,kw |
| 6 | #4 AND #5 |
| 7 | Limits date from Jan 2010 to Dec 2022 |

1. **CINAHL**

| **Component** | **Search terms** |
| --- | --- |
| 1 | “Perinatal Mortality” or stillbirth or “fetal death” or “infant mortality” |
| 2 | (perinat* or f?etal or f?etus* or infant* or neonat*) N5 (death* or mortalit* or demise) |
| 3 | stillbirt* or stillborn* or “adverse birth outcome*” or “pregnancy outcome*” or “perinatal outcome*” |
| **4** | **1 Or 2 OR 3** |
| 5 | “eastern Africa*” or Burundi or Burundian or Comoros or Comorian or Djibouti or Djiboutian or Eritrea or Eritrean or Ethiopia or Ethiopian or Kenya or Kenyan or Madagascar or Malagasy or Madagascan or Malawi or Malawian or Mauritius or Mauritian or Mozambique or Mozambican or Rwanda or Rwandan or Seychelles or Seychellois or Somalia or Somalis or Somalian or Somaliland or “South Sudan” or Sudan or Sudanese or Tanzania or Tanzanian or Uganda or Ugandan or Zambia or Zambian or Zimbabwe or Zimbabwean |
| **7** | **4 AND 5** |
| **8** | **Limiters** - Published Date: 20100101-20221231 |

1. **African Index Medicus**

| **Component** | **Search terms** |
| --- | --- |
| 1 | 1 Or 2 OR 3  “perinatal mortality” OR stillb* OR “fetal death” OR “infant mortality” OR "perinat* death" OR "perinat* mortali*" OR "fetal death*" OR "fetal mortal*" OR "fetal demise" OR "foetus* death*" OR "foetus* mortal*" OR "infant* death*" OR "infant mortal*" OR "neonat* death*" OR "neonat* mortal*" AND ( pais_assunto:("africa")) AND (year_cluster:[2010 TO 2022]) |
| 2 | ( pais_assunto:("africa")) |
| 3 | (year_cluster:[2010 TO 2022]) |
| 4 | 1 AND 2 AND 3 |

**Supplementary Appendix 4: Characteristics of Studies on perinatal mortality in East Africa.**

| **Author,**  **Year** | **Country** | **Study design** | **Scope** | **Study setting** | **Mortality reported** | **Stillbirth definition** | **Multiple gestation  Included** | **PM** | **ENM** | **SB** | **Timing of Stillbirths reported** |
| --- | --- | --- | --- | --- | --- | --- | --- | --- | --- | --- | --- |
| Abebe H, 2021 | Ethiopia | Case-control | Sub-national | Health facility | Stillbirth | Fetal death >= 28 weeks | Yes |  |  | 138 | No |
| Adane A A, 2014 | Ethiopia | Cross-sectional | Sub-national | Health facility | Stillbirth | Fetal death >= 28 weeks | Yes |  |  | 34 | No |
| Addila A E, 2021 | Ethiopia | Cohort | Sub-national | Health facility | Stillbirth | Fetal death >= 28 weeks | No |  |  | 72 | No |
| Ajaari J, 2012 | Tanzania | Cross-sectional | Sub-national | Population-based | Early neonatal mortality | - | NS |  | 111 |  | _ |
| Akombi BJ, 2018 | Burundi, Kenya, Rwanda,  Tanzania and Uganda | Cross-sectional | National | Population-based | Stillbirth | Fetal death >= 7months | NS |  |  | 1002 | No |
| Alene A G, 2022 | Ethiopia | Cohort | Sub-national | Health facility | Perinatal mortality | Fetal death >= 28 weeks | NS | 27 |  |  | No |
| Alsammani MA, 2021 | Sudan | Cross-sectional | Sub-national | Health facility | Stillbirth | Fetal death >= 24 weeks | Yes |  |  | 285 | Yes |
| Aminu M, 2019 | Kenya | Case series | Sub-national | Health facility | Stillbirth | Fetal death >= 28 weeks | Yes |  |  | 414 | Yes |
|  | Malawi |  |  |  |  |  |  |  |  | 356 |  |
|  | Zimbabwe |  |  |  |  |  |  |  |  | 445 |  |
| Arach AAO, 2021 | Uganda | Cohort | Sub-national | Population-based | Perinatal mortality | Fetal death >= 28 weeks | Yes | 81 | 44 | 37 | Yes |
| Asiki G, 2015 | Uganda | Cross-sectional | Sub-national | Population-based | Stillbirth | Fetal death >= 28 weeks | NS |  |  | 61 | No |
| Assefa N, 2016 | Ethiopia | Cohort | Sub-national | Population-based | Early neonatal mortality | - | NS |  | 248 |  | _ |
| Bakar R R, 2019 | Tanzania | Cohort | Sub-national | Health facility | Perinatal mortality | Fetal death >= 28 weeks | No | 40 | 14 | 26 | No |
| AMANHI, 2018 | Kenya, | Cohort | Sub-national | Population-based | Stillbirth | Fetal death >= 28 weeks | NS |  | 287 | 233 | Yes |
|  | Tanzania |  |  |  |  |  |  |  | 409 | 621 |  |
|  | Zambia |  |  |  |  |  |  |  | 279 | 441 |  |
| Berhe T, 2019 | Ethiopia | Cross-sectional | Sub-national | Health facility | Stillbirth | Fetal death >= 28 weeks | Yes |  |  | 21 | No |
| Berhe AK, 2020 | Ethiopia | Cohort | Sub-national | Population-based | Perinatal mortality | Fetal death >= 28 weeks | Yes |  |  | 35 |  |
| Berhie KA, 2016 | Ethiopia | Cross-sectional | National | Population-based | Stillbirth | Fetal death >= 7months | NS |  |  | 320 | No |
| Beyene T, 2022 | Ethiopia | Cross-sectional | Sub-national | Health facility | Perinatal mortality | Fetal death >= 28 weeks | No | 1129 | 377 | 752 | No |
| Burundi-DHS, 2017 | Burundi | Cross-sectional | National | Population-based | Perinatal mortality | Fetal death >= 7months | NS | 528 | 215 | 313 | No |
| Chan GJ, 2022 | Ethiopia | Cohort | Sub-national | Population-based | Perinatal mortality | Fetal death >=28 weeks | Yes | 107 | 48 | 59 | No |
| Chekol A, 2011 | Ethiopia | Cross-sectional | Sub-national | Health facility | Perinatal mortality | Fetal death >=28 weeks | NS | 69 | 13 | 56 | No |
| Chuwa F S, 2017 | Tanzania | Cohort | Sub-national | Health facility | Stillbirth | Fetal death >= 28 weeks or weight >1000 gram | No |  |  | 1659 | Yes |
| Dagne HM, 2021 | Ethiopia | Case-control | Sub-national | Health facility | Stillbirth | Fetal death >=28 weeks or at least 1000gm | Yes |  |  | 134 | Yes |
| Dasa TT, 2022 | Ethiopia | Cohort | Sub-national | Health facility | Stillbirth | Fetal death >= 28 weeks | No |  |  | 50 | No |
| Debelew GT, 2020 | Ethiopia | Case-control | Sub-national | Population-based | Perinatal mortality | Fetal death >=28 weeks | Yes | 120 | 73 | 47 | No |
| Debelo BT, 2020 | Ethiopia | Cohort | Sub-national | Health facility | Stillbirth | Fetal death >=28 weeks | No |  |  | 59 | No |
| Demise A, 2015 | Ethiopia | Cross-sectional | Sub-national | Health facility | Perinatal mortality | Fetal death >= 28 weeks | NS | 61 | 31 | 30 | No |
| Dessu S, 2020 | Ethiopia | Cross-sectional | Sub-national | Health facility | Perinatal mortality | Fetal death >=28 weeks | Yes | 230 |  |  | No |
| Desta B, 2016 | Ethiopia | Cohort | Sub-national | Population-based | Early neonatal mortality | - | Yes |  | 144 |  | _ |
| Dheresa M, 2022 | Ethiopia | Cohort | Sub-national | Population-based | Perinatal mortality | Fetal death >= 28 weeks | NS | 783 | 347 | 436 | No |
| Dube K, 2021 | Zimbabwe | Cross-sectional | Sub-national | Health facility | Stillbirth | Fetal death >= 28 weeks or weight >=1000gm | No |  |  | 43 | Yes |
| Ethiopia-DHS, 2012 | Ethiopia | Cross-sectional | National | Population-based | Perinatal mortality | Fetal death >= 7months | NS | 551 | 347 | 204 | No |
| Ethiopia-DHS, 2016 | Ethiopia | Cross-sectional | National | Population-based | Perinatal mortality | Fetal death >= 7months | NS | 366 | 236 | 130 | No |
| Fikre R, 2021 | Ethiopia | Case-control | Sub-national | Health facility | Stillbirth | Fetal death >=28 weeks, with a weight of 1000gm | Yes |  |  | 106 | Yes |
| Gebreslasie KZ, 2020 | Ethiopia | Cross-sectional | Sub-national | Health facility | Stillbirth | Fetal death >=28 weeks | NS |  |  | 23 | No |
| Geda A, 2021 | Ethiopia | Case-control | Sub-national | Health facility | Perinatal mortality | >=28 weeks, and/or >500gm | No |  |  | 90 | No |
| Ghosh R, 2022 | Uganda and  Kenya | Cohort | Sub-national | Health facility | Stillbirth | Fetal death >=20 weeks | No |  |  | 3734 | Yes |
| Girma D, 2022 | Ethiopia | Cross-sectional | Sub-national | Population-based | Perinatal mortality | Fetal death >= 7months | Yes | 182 |  |  | No |
| Gizaw W, 2021 | Ethiopia | Case-control | Sub-national | Health facility | Stillbirth | Fetal death >=28 weeks | NS |  |  | 69 | No |
| Gurmu L, 2022 | Ethiopia | Cohort | Sub-national | Health facility | Perinatal mortality | Fetal death >=28 weeks | No | 4 |  | NR | No |
| Gwako GN, 2020 | Kenya | Case-control | Sub-national | Health facility | Stillbirth | Fetal death >=28 weeks | NS |  |  | 720 | Yes |
| Habte M B, 2021 | Ethiopia | Case-control | Sub-national | Health facility | Stillbirth | Fetal death >=28 weeks | NS |  |  | 248 | No |
| Harrison M S, 2021 | Ethiopia | Cross-sectional | Sub-national | Health facility | Stillbirth | Fetal death >=28 weeks | Yes |  |  | 43 | Yes |
| Housseine N, 2021 | Tanzania | Cross-sectional | Sub-national | Health facility | Perinatal mortality | weight >1000 gm | Yes | 661 | 222 | 413 | Yes |
| Jaleta DD, 2021 | Ethiopia | Cohort | Sub-national | Health facility | Perinatal mortality | Fetal death >= 28 weeks | Yes | 87 | 37 | 50 | No |
| Jena B H, 2021 | Ethiopia | Cohort | Sub-national | Population-based | Stillbirth | Fetal death >=28 weeks or weight >1000gm | NS |  |  | 38 | Yes |
| Jena B H, 2022 | Ethiopia | Cohort | Sub-national | Population-based | Perinatal mortality | >=28 weeks | NS | 47 |  |  | Yes |
| Kamala BA, 2018 | Tanzania | Cross-sectional | Sub-national | Health facility | Stillbirth | Fetal death >=28 weeks | Yes |  | 704 (Very early neonatal mortality) | 2698 | No |
| Kananura RM, 2021 | Uganda | Cross-sectional | Sub-national | Population-based | Perinatal mortality | Fetal death >=22 weeks | Yes | 333 |  |  | No |
| Kassahun EA, 2019 | Ethiopia | Cross-sectional | Sub-national | Health facility | Stillbirth | Fetal death >=28 weeks | NS |  |  | 36 | No |
| Kebede E, 2021 | Ethiopia | Case-control | Sub-national | Health facility | Perinatal mortality | >=28 weeks | Yes | 366 |  |  | Yes |
| Kenya-DHS, 2015 | Kenya | Cross-sectional | National | Population-based | Perinatal mortality | Fetal death >= 7months | NS | 272 | 146 | 126 | No |
| Konje E T, 2020 | Tanzania | Cohort | Sub-national | Population-based | Perinatal mortality | Fetal death after the third trimester (>=28 weeks) | No | 29 | 14 | 15 | No |
| Kujala S, 2017 | Uganda | Cross-sectional | Sub-national | Population-based | Stillbirth | Fetal death >=28 weeks | Yes |  |  | 515 | No |
| Kunkel M, 2019 | Kenya | Cohort | Sub-national | Population-based | Perinatal mortality | Fetal death >=20 weeks | Yes | 1085 | 385 | 700 | No |
| Lakew D, 2017 | Ethiopia | Cross-sectional | Sub-national | Population-based | Stillbirth | Fetal death >= 28 weeks | NS |  |  | 218 | No |
| Lema G, 2020 | Tanzania | Case-control | Sub-national | Health facility | Stillbirth | Fetal death >= 28 weeks | NS |  |  | 98 | Yes |
| Limaso A A, 2020 | Ethiopia | Cohort study | Sub-national | Population-based | Early neonatal mortality | - | Yes |  | 15 | 14 | No |
| Liyew A D, 2021 | Ethiopia | Case-control | Sub-national | Health facility | Stillbirth | Fetal death >=28 weeks Or >=1000grams | NS |  |  | 117 | No |
| Lohela TJ, 2012 | Malawi | Cross-sectional | National | Population-based | Early neonatal mortality | - | No |  | 198 |  | - |
|  | Zambia |  |  |  |  |  |  |  | 96 |  |  |
| Lolaso T, 2021 | Ethiopia | Cross-sectional | Sub-national | Health facility | Stillbirth | Fetal death >=28 weeks | No |  |  | 179 | No |
| Lund S, 2014 | Tanzania | Cluster randomized trial | Sub-national | Health facility | Perinatal mortality | >=28 weeks Or weight >=1000gm | Yes | 69 | 15 | 54 | Yes |
| Madagascar-DHS, 2022 | Madagascar | Cross-sectional | National | Population-based | Perinatal mortality | Fetal death >= 7months | NS | 460 | 252 | 208 | No |
| Mahande MJ, 2016 | Tanzania | Cohort | Sub-national | Health facility | Perinatal mortality | Fetal death >=28 weeks | No | 557 |  |  | No |
| Makuluni R, 2021 | Malawi | Cross-sectional | Sub-national | Health facility | Stillbirth | Fetal death >=28 weeks | NS |  |  | 67 | Yes |
| Malawi-DHS, 2017 | Malawi | Cross-sectional | National | Population-based | Perinatal mortality | Fetal death >= 7months | NS | 614 | 378 | 236 | No |
| Mangu C D, 2021 | Tanzania | Cross-sectional | Sub-national | Health facility | Early neonatal mortality | - | NS |  | 23590 |  | - |
| Mboya I B, 2020 | Tanzania | Cohort | Sub-national | Health facility | Perinatal mortality | Fetal death >= 7months | No | 2108 |  |  | No |
| McKinnon B, 2014 | Ethiopia | Cross-sectional | National | Population-based | Early neonatal mortality | - | Yes |  | 231 |  | - |
| Mengesha HG, 2017 | Ethiopia | Cohort study | Sub-national | Health facility | Early neonatal mortality | - | NS |  | 51 |  | - |
| Mengistu S, 2022 | Ethiopia | Cross-sectional | Sub-national | Health facility | Stillbirth | Fetal death >=28 weeks and birth weight >= 1000 gm | NS |  |  | 81 | No |
| Mersha A, 2020 | Ethiopia | Cohort | Sub-national | Population-based | Stillbirth | Fetal death >=28 weeks | NS |  |  | 25 | No |
| Mmbaga BT, 2012 | Tanzania | Cross-sectional | Sub-national | Health facility | Perinatal mortality | 500 grams or more | Yes | 1958 | 739 | 1219 | Yes |
| Mohammed-Ahmed A, 2022 | Ethiopia | Cross-sectional | Sub-national | Health facility | Stillbirth | Fetal death >=28 weeks with a birth weight of 1000g or more | NS |  |  | 42 | No |
| Musafili A, 2015 | Rwanda | Case-control | Sub-national | Health facility | Perinatal mortality | Fetal death >=22 weeks or at least 500gm | No | 269 | 100 | 169 | Yes |
| Musafili A, 2017 | Rwanda | Cross-sectional | Sub-national | Health facility | Perinatal mortality | Fetal death >=22 weeks or weighing 500gm or more | Yes | 269 | 100 | 169 | Yes |
| Nakibuuka VK, 2012 | Uganda | Case series | Sub-national | Health facility | Perinatal mortality | Fetal death >=28 weeks or >1000gm or ht >=25cm | Yes | 350 |  | 120 | Yes |
| Nankabirwa V, 2011 | Uganda | Cohort | Sub-national | Population-based | Perinatal mortality | Fetal death >=28 weeks | Yes | 34 | 18 | 16 | No |
| Ngwenya S, 2018 | Zimbabwe | Cross-sectional | Sub-national | Health facility | Stillbirth | Fetal death >=28 weeks Or >500gm | Yes |  |  | 268 | Yes |
| Roro EM, 2018 | Ethiopia | Case-control | Sub-national | Population-based | Perinatal mortality | Fetal death >=28 weeks or after 7 completed months | Yes | 73 | 47 | 26 | No |
| Rwanda-DHS, 2021 | Rwanda | Cross-sectional | National | Population-based | Perinatal mortality | Fetal death >= 7months | NS | 235 | 124 | 111 | No |
| Schmiegelow C, 2012 | Tanzania | Cohort study | Sub-national | Health facility | Perinatal mortality | Fetal death >=28 weeks | Yes | 46 | 21 | 25 | Yes |
| Seyoum E, 2021 | Ethiopia | Cross-sectional | Sub-national | Health facility | Perinatal mortality | Fetal death >=28 weeks | NS | 84 | 21 | 63 | No |
| Shiferaw K, 2022 | Ethiopia | Cross-sectional | Sub-national | Population-based | Early neonatal mortality | - | No |  | 47 |  | _ |
| Sifer SD, 2020 | Ethiopia | Cohort study | Sub-national | Health facility | Perinatal mortality | Fetal death >=28 weeks | Yes | 74 |  |  | No |
| Tadese M, 2022 | Ethiopia | Cross-sectional | Sub-national | Health facility | Stillbirth | Fetal death >=28 weeks | Yes |  |  | 332 | No |
| Tanzania-DHS, 2016 | Tanzania | Cross-sectional | National | Population-based | Perinatal mortality | Fetal death >= 7months | NS | 401 | 214 | 187 | No |
| Tesema GA, 2020 | Ethiopia | Cross-sectional | National | Population-based | Stillbirth | Fetal death >= 7months | Yes |  |  | 105 | No |
| Tesfaye N, 2022 | Ethiopia | Cross-sectional | National | Health facility | Early neonatal mortality | Fetal death >=28 weeks | NS | 3814 | 2397 | 1417 | Yes |
| Turnbull E, 2011 | Zambia | Cross-sectional | Sub-national | Population-based | Stillbirth | Fetal death >=28 weeks | Yes |  |  | 50 | No |
| Twabi HS, 2020 | Malawi | Cross-sectional | National | Population-based | Perinatal mortality | Fetal death >= 7months | NS |  |  |  | No |
| Uganda-DHS, 2018 | Uganda | Cross-sectional | National | Population-based | Perinatal mortality | Fetal death >= 7months | NS | 581 | 330 | 251 | No |
| Woldeamanuel BT, 2019 | Ethiopia | Cross-sectional | Sub-national | Population-based | Perinatal mortality | Fetal death >= 7months | Yes | 170 | 111 | 59 | No |
| Worede DT, 2019 | Ethiopia | Case-control | Sub-national | Health facility | Stillbirth | Fetal death >=28 weeks | Yes |  |  | 84 | No |
| Yadeta TA, 2020 | Ethiopia | Cross-sectional | National | Population-based | Perinatal mortality | Fetal death >= 7months | NS | 350 | 235 | 115 | No |
| Yirgu R, 2016 | Ethiopia | Case-control | Sub-national | Population-based | Perinatal mortality | Fetal death >=28 weeks | Yes | 102 | 45 | 57 | No |
| Zambia-DHS, 2014 | Zambia | Cross-sectional | National | Population-based | Perinatal mortality | Fetal death >= 7months | Yes | 427 | 247 | 180 | No |
| Zambia-DHS, 2019 | Zambia | Cross-sectional | National | Population-based | Perinatal mortality | Fetal death >= 7months | Yes | 325 | 206 | 119 | No |
| Zimbabwe-DHS, 2016 | Zimbabwe | Cross-sectional | National | Population-based | Perinatal mortality | Fetal death >= 7months | NS | 217 | 140 | 77 | No |
| Zuanna TD, 2021 | Ethiopia | Case-control | Sub-national | Health facility | Perinatal mortality | Fetal death >= 28 weeks or >1000gm | Yes | 1175 |  |  | No |

**Supplementary Appendix 5: Quality assessment and risk of bias**

Table S5.1: Quality assessment of cross-sectional studies on perinatal mortality in East Africa using Joanna Briggs Institute (JBI) tool.

Table S5.2: Quality assessment of cohort studies on perinatal mortality in East Africa using JBI tool.

Table S5.3: Quality assessment of case-control studies on perinatal mortality in East Africa using JBI tool.

Table S5.4: Quality assessment of case-series studies on perinatal mortality in East Africa using JBI tool.

Table S5.5: Quality assessment of community-based trial studies on perinatal mortality in East Africa using JBI tool.

Table S5.6: The risk of bias assessment for studies included in the pooled perinatal mortality rate in East Africa (Hoy et al., 2012).

| **Authors / Year** | **External validity** | | | | | | | | **Internal validity** | | | | | | | | | | |  | **Summary** |
| --- | --- | --- | --- | --- | --- | --- | --- | --- | --- | --- | --- | --- | --- | --- | --- | --- | --- | --- | --- | --- | --- |
|  | **Q1** | | **Q2** | | **Q3** | | **Q4** | | **Q5** | | **Q6** | | **Q7** | | **Q8** | | **Q9** | | **Q10** | |  |
|  | **Y** | **N** | **Y** | **N** | **Y** | **N** | **Y** | **N** | **Y** | **N** | **Y** | **N** | **Y** | **N** | **Y** | **N** | **Y** | **N** | **Y** | **N** |  |
| Arach 2021 |  | N | Y |  | Y |  | Y |  | Y |  | Y |  |  | N | Y |  | Y |  | Y |  | 8 |
| Bakar 2019 | Y |  |  | N |  | N | Y |  | Y |  | Y |  | Y |  | Y |  | Y |  | Y |  | 8 |
| AMANHI, 2018 | Y |  | Y |  | Y |  | Y |  | Y |  | Y |  |  | N | Y |  | Y |  | Y |  | 9 |
| Beyene 2022 |  | N |  | N | Y |  | Y |  |  | N | Y |  | Y |  | Y |  |  | N |  | N | 6 |
| Brundi DHS 2017 | Y |  | Y |  | Y |  | Y |  | Y |  | Y |  |  | N | Y |  | Y |  | Y |  | 9 |
| Chan 2022 | N |  | Y |  | Y |  | Y |  | Y |  | Y |  |  | N | Y |  | Y |  | Y |  | 8 |
| Chekol 2011 | Y |  |  | N |  | N | Y |  | Y |  | Y |  |  | N | Y |  |  | N |  | N | 5 |
| Debelew 2020 | Y |  | Y |  | Y |  | Y |  | Y |  | Y |  |  | N | Y |  | Y |  | Y |  | 9 |
| Demise 2015 |  | N |  | N | Y |  | Y |  |  | N | Y |  | Y |  | Y |  | Y |  | Y |  | 7 |
| Dessu 2020 | Y |  |  | N | Y |  | Y |  | Y |  | Y |  | Y |  |  | N | Y |  | Y |  | 8 |
| Dheresa 2022 | Y |  | Y |  | Y |  | Y |  | Y |  | Y |  |  | N | Y |  | Y |  | Y |  | 9 |
| Ethiopia DHS 2012 | Y |  | Y |  | Y |  | Y |  | Y |  | Y |  |  | N | Y |  | Y |  | Y |  | 9 |
| Ethiopia DHS 2017 | Y |  | Y |  | Y |  | Y |  | Y |  | Y |  |  | N | Y |  | Y |  | Y |  | 9 |
| Girma 2022 | Y |  | Y |  | Y |  | Y |  | Y |  | Y |  |  | N | Y |  | Y |  | Y |  | 9 |
| Jena 2022 |  | N | Y |  | Y |  | Y |  | Y |  | Y |  |  | N | Y |  | Y |  | Y |  | 8 |
| Kenya DHS 2015 | Y |  | Y |  | Y |  | Y |  | Y |  | Y |  |  | N | Y |  | Y |  | Y |  | 9 |
| Konje 2020 |  | N | Y |  | Y |  |  | N | Y |  | Y |  |  | N |  | N | Y |  | Y |  | 6 |
| Lund 2014 | Y |  |  | N | Y |  | Y |  | Y |  | Y |  |  | N |  | N | Y |  | Y |  | 7 |
| Madagascar DHS 2022 | Y |  | Y |  | Y |  | Y |  | Y |  | Y |  |  | N | Y |  | Y |  | Y |  | 9 |
| Malawi DHS 2017 | Y |  | Y |  | Y |  | Y |  | Y |  | Y |  |  | N | Y |  | Y |  | Y |  | 9 |
| Mboya 2020 | Y |  |  | N | Y |  | Y |  |  | N | Y |  | Y |  | Y |  | Y |  | Y |  | 8 |
| Nakibuuka 2012 |  | N |  | N | Y |  | Y |  | Y |  | Y |  | Y |  | Y |  | Y |  | Y |  | 8 |
| Nankabirwa 2011 |  | N | Y |  | Y |  | Y |  | Y |  | Y |  |  | N | Y |  | Y |  | Y |  | 8 |
| Roro 2018 |  | N | Y |  | Y |  | Y |  | Y |  | Y |  |  | N | Y |  | Y |  | Y |  | 8 |
| Rwanda DHS 2021 | Y |  | Y |  | Y |  | Y |  | Y |  | Y |  |  | N | Y |  | Y |  | Y |  | 9 |
| Schmiegelow 2012 |  | N |  | N |  | N | Y |  | Y |  | Y |  | Y |  | Y |  | Y |  | Y |  | 7 |
| Seyoum 2021 | Y |  |  | N | Y |  | Y |  |  | N | Y |  | Y |  | Y |  |  | N |  | N | 6 |
| Tanzania DHS 2016 | Y |  | Y |  | Y |  | Y |  | Y |  | Y |  |  | N | Y |  | Y |  | Y |  | 9 |
| Uganda DHS 2018 | Y |  | Y |  | Y |  | Y |  | Y |  | Y |  |  | N | Y |  | Y |  | Y |  | 9 |
| Woldeamanuel 2019 | Y |  | Y |  | Y |  | Y |  | Y |  | Y |  |  | N | Y |  | Y |  | Y |  | 9 |
| Yirgu 2016 |  | N | Y |  | Y |  | Y |  | Y |  | Y |  |  | N | Y |  | Y |  | Y |  | 8 |
| Zambia DHS 2015 | Y |  | Y |  | Y |  | Y |  | Y |  | Y |  |  | N | Y |  | Y |  | Y |  | 9 |
| Zambia DHS 2020 | Y |  | Y |  | Y |  | Y |  | Y |  | Y |  |  | N | Y |  | Y |  | Y |  | 9 |
| Zimbabwe DHS 2016 | Y |  | Y |  | Y |  | Y |  | Y |  | Y |  |  | N | Y |  | Y |  | Y |  | 9 |

**Items used for risk of bias assessment for prevalence studies**

**External validity**

Q1. Was the study target population a close representation of the national pregnant population

in relation to relevant variables?

Q2. Was the sampling frame a true or close representation of the target population?

Q3. Was some form of random selection used to select the sample or was a census undertaken?

Q4. Was the likelihood of non-participation bias minimal?

**Internal validity**

Q5. Were data collected directly from the subjects?

Q6. Were acceptable case definitions used for perinatal mortality or/and SB/END used?

Q7. Was a reliable and accepted method of measuring perinatal mortality used?

Q8. Was the same mode of data collection used for all subjects?

Q9. Was the length of the shortest prevalence period for the parameter of interest appropriate?

Q10. Were the numerator(s) and denominator(s) for the parameter of interest appropriate?

Figure S5.1: Risk of bias assessment by Risk Of Bias In Non-randomized Studies - of Exposures (ROBINS-E).


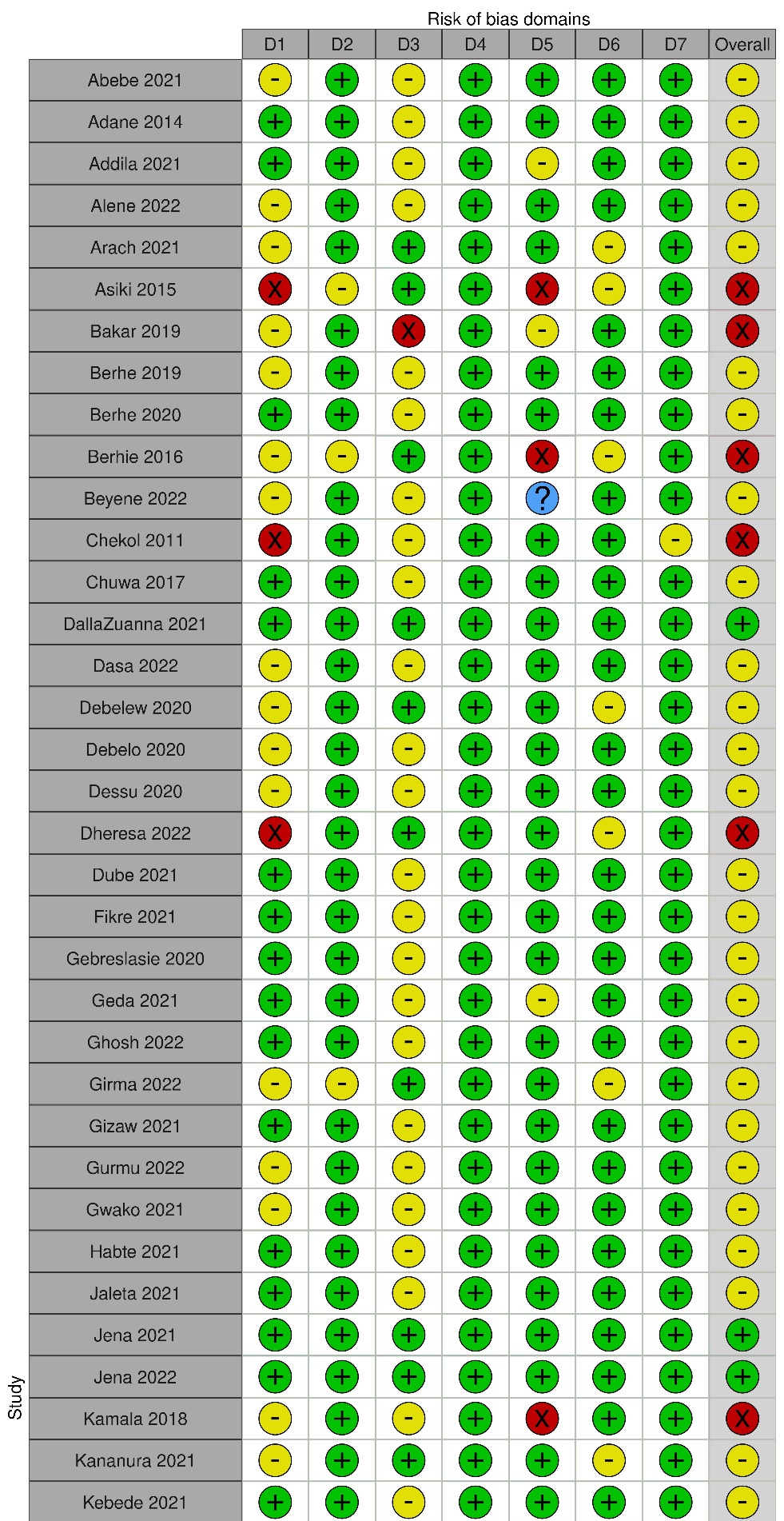


**Continued**


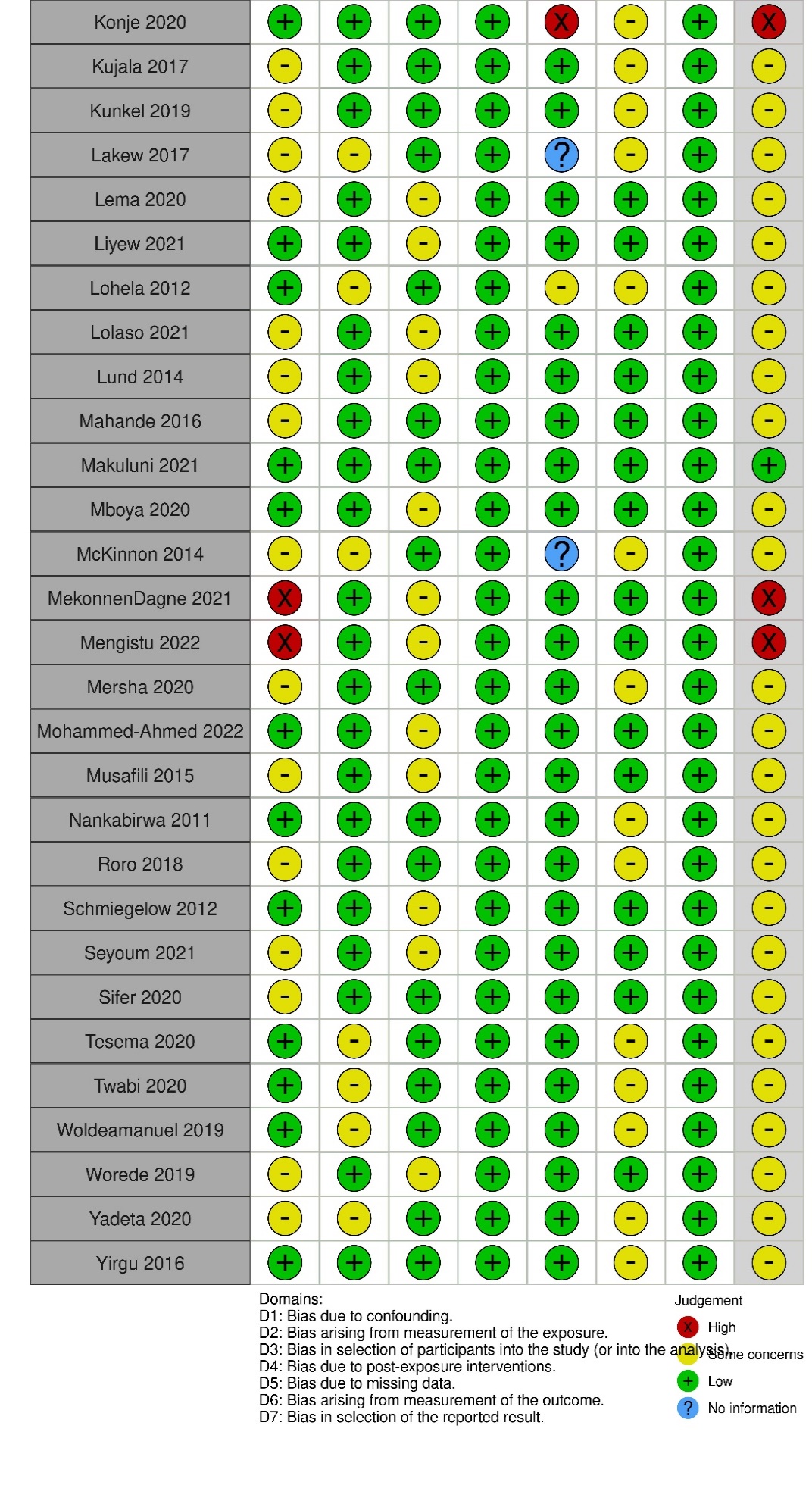


Figure S5.2: Overall summary of the risk of bias assessment using the Risk Of Bias In Non-randomized Studies - of Exposures (ROBINS-E).


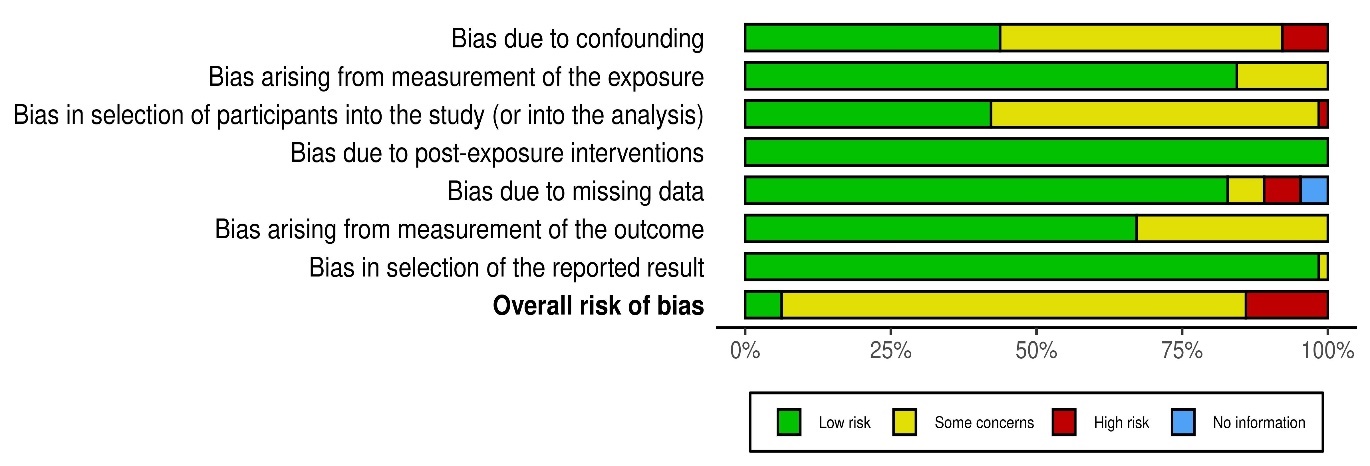


**Supplementary Appendix 6: Pooled stillbirth rate and early neonatal mortality rate in East Africa.**

Figure S6.1: Meta-analysis showing pooled stillbirth rates in population-based settings and health facilities in East Africa (2010 – 2022).


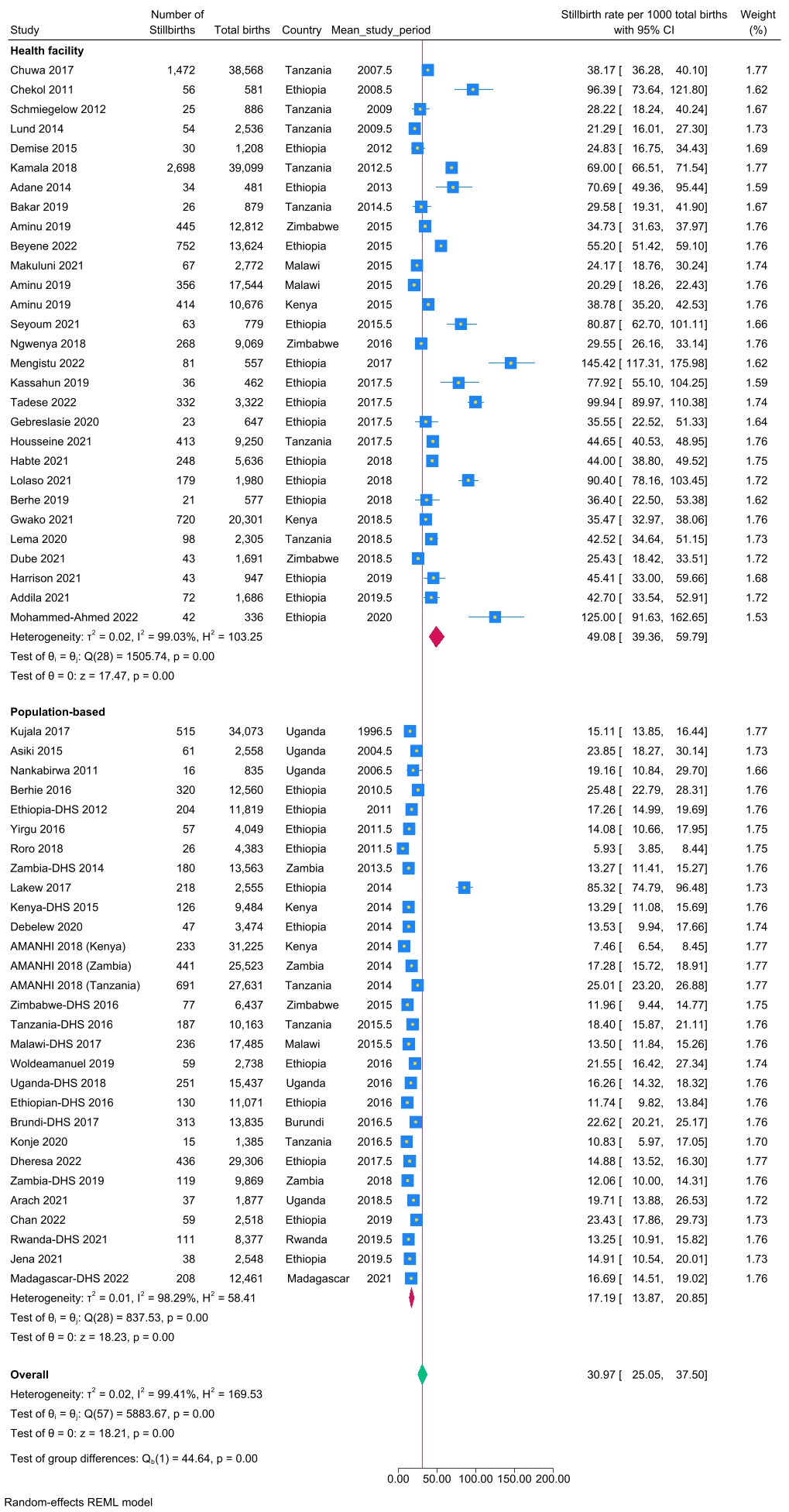


Figure S6.2: Meta-analysis showing pooled early neonatal mortality rates in population-based settings and health facilities in East Africa (2010 – 2022).


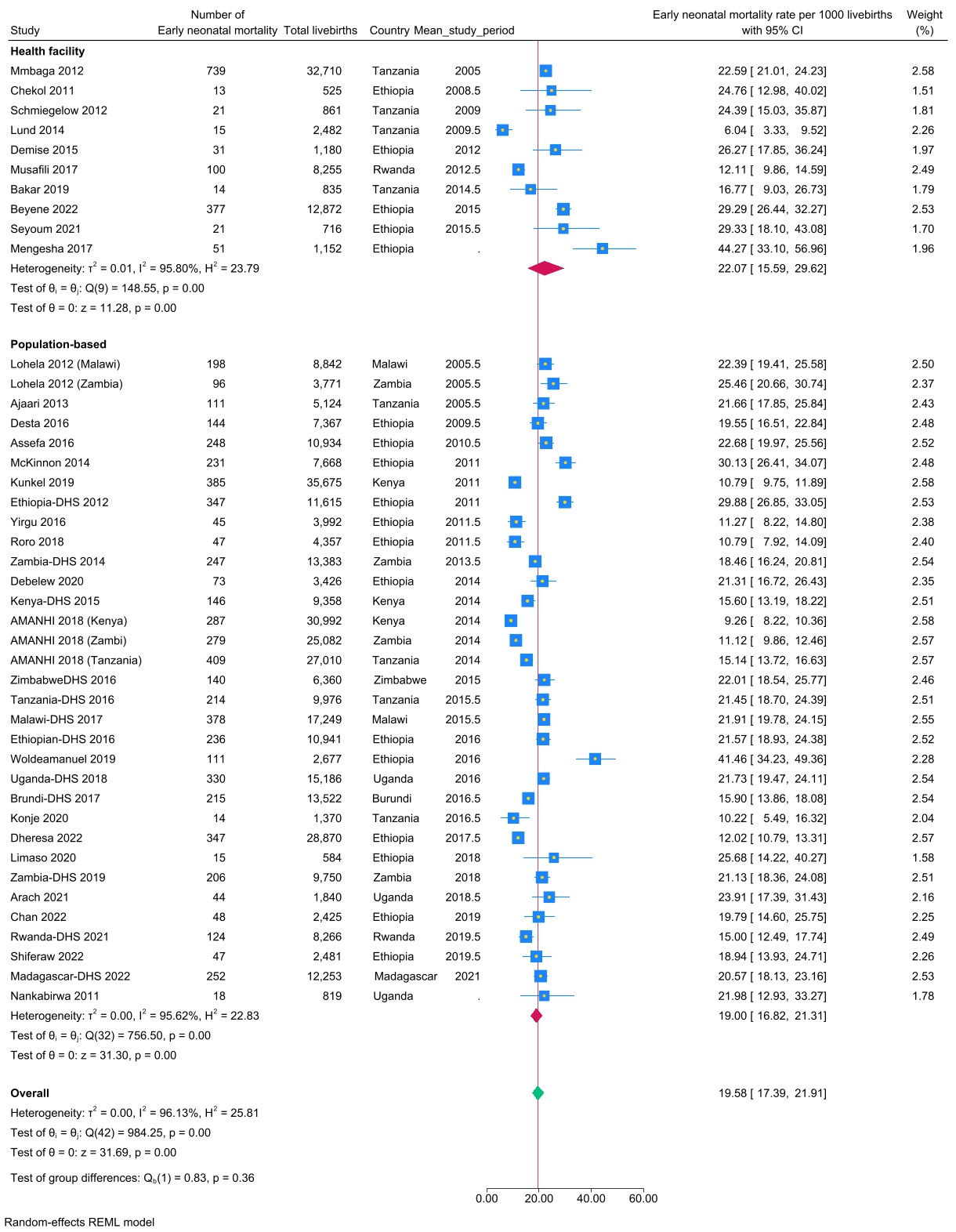


**Supplementary Appendix 7: Galbraith plot studies included in population-based pooled perinatal mortality in East Africa.**


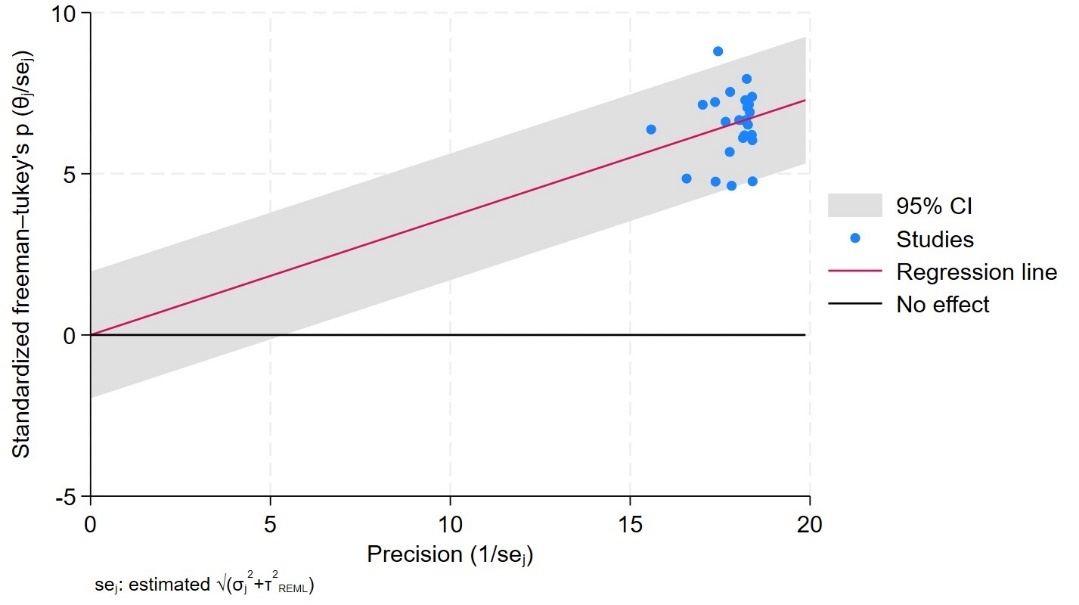


**Supplementary Appendix 8: Sub-group analysis**

The subgroup analysis among population-based studies was done based on the scope of the study, study period (data collected before or after the implementation of ENAP - 2014), economy, risk of bias, study design, place of residence, and country. From the sub-group analysis, nationwide studies, studies conducted after the implementation of ENAP (after 2014), cross-sectional studies, and studies from lower-income countries showed higher perinatal mortality rates. The perinatal mortality rate was higher in Uganda at 38.5(35.0, 42.2), followed by Burundi at 38.1 (35.0, 41.4) and Madagascar at 36.9 (33.7, 40.3).

Table S8.1: Subgroup analysis of Population-based studies on perinatal mortality in East Africa.

| **Subgroups** | | **Number of**  **studies** | **PMR (95% CI)** | **Heterogeneity** | |
| --- | --- | --- | --- | --- | --- |
|  |  |  |  | **I^2^** | **P-value** |
| Scope | National | 12 | 35.05 (32.29, 37.93) | 88.18 % | < 0.01 |
|  | Sub-national | 24 | 31.49 (25.05, 38.63) | 97.91 % | < 0.01 |
| ENAP | Before ENAP (Study period before 2015) | 10 | 29.90 (23.92, 36.52) | 97.62 % | < 0.01 |
|  | After ENAP (Study period on/ after 2015) | 16 | 35.20 (30.70, 39.99) | 95.62 % | < 0.01 |
| Economy | Low-income | 17 | 34.93 (29.90, 40.33) | 96.46% | < 0.01 |
|  | Lower-middle income | 9 | 29.94 (24.93, 35.40) | 96.72 % | < 0.01 |
| Risk of bias | High | 1 | 20.94 (14.00, 29.20) | - | < 0.01 |
|  | Moderate | 6 | 29.65 (20.31, 40.66) | 92.97 % | < 0.01 |
|  | Low | 19 | 34.74 (30.82, 38.88) | 96.88 % | < 0.01 |
| Study design | Cross-sectional | 14 | 37.07 (33.17, 41.18) | 94.03 % | < 0.01 |
|  | Cohort | 9 | 29.65 (22.93, 37.19) | 97.85 % | < 0.01 |
|  | Case-control | 3 | 24.89 (15.75, 36.03) | 92.32 % | < 0.01 |
| Place of residence | Both urban and rural | 17 | 36.33 (32.80, 40.04) | 93.91 % | < 0.01 |
|  | Rural | 8 | 28.12 (20.93, 36.33) | 97.81 % | < 0.01 |
|  | Urban | 1 | 18.45 (13.56, 24.06) | - | - |
| Country | Burundi | 1 | 38.06 (35.03, 41.42) | - | - |
|  | Ethiopia | 10 | 33.73 (25.73, 42.78) | 97.38 % | < 0.01 |
|  | Kenya | 2 | 22.20 (11.94, 35.52) | 97.94 % | < 0.01 |
|  | Madagascar | 1 | 36.92 (33.67, 40.30) | - | - |
|  | Malawi | 1 | 35.12 (32.44, 37.90) | - | - |
|  | Rwanda | 1 | 28.05 (24.62, 31.70) | - | - |
|  | Tanzania | 3 | 33.57 (22.66, 46.51) | 96.40 % | < 0.01 |
|  | Uganda | 3 | 38.52 (34.97, 42.24) | 11.57 % | < 0.01 |
|  | Zambia | 3 | 30.61 (27.80, 33.54) | 68.92 % | < 0.01 |
|  | Zimbabwe | 1 | 33.71 (29.44, 38.26) | - | - |

Sub-groups with high perinatal mortality rates among health facility-based studies included studies conducted after ENAP (2014), mean study period after 2014) 104.2 per 1000 births (79.3, 132.1), cross-sectional studies 94.8 per 1000 births (68.1, 125.4, studies from low-income countries 87.8 per 1000 births (63.4, 115.6), studies with high risk of bias 100.1 per 1000 births (78.5, 124.0), and studies from referral health facilities 80.6 per 1000 births (56.8, 108.1).

Table S8.2: Subgroup analysis of health facility-based studies on perinatal mortality in East Africa.

| **Subgroups** | | **Number of**  **studies** | **PMR (95% CI)** | **Heterogeneity** | |
| --- | --- | --- | --- | --- | --- |
|  |  |  |  | **I^2^** | **P-value** |
| ENAP | Before ENAP (Study period before 2015) | 7 | 52.97 (36.08, 72.85) | 97.6% | < 0.01 |
|  | After ENAP (Study period on/ after 2015) | 3 | 104.17 (79.25, 132.06) | 92.2% | < 0.01 |
| Economy | Low-income | 6 | 87.78 (63.44, 115.62) | 97.3% | < 0.01 |
|  | Lower-middle income | 4 | 40.19 (30.26, 51.45) | 86.7% | < 0.01 |
| Risk of bias | High | 3 | 100.07 (78.46, 123.97) | 81.9% | < 0.01 |
|  | Moderate | 7 | 54.45 (35.50, 77.13) | 98.27% | < 0.01 |
| Study design | Cross-sectional | 5 | 94.79 (68.06, 125.38) | 95.53% | < 0.01 |
|  | Cohort | 3 | 43.44 (37.78, 49.47) | 32.7% | 0.26 |
|  | Case series | 1 | 27.21 (21.21, 33.92) | - | < 0.01 |
|  | Cluster randomised trial | 1 | 57.94 (52.18, 63.97) | - | < 0.01 |
| Type of facility | Referral | 5 | 80.6 (56.8, 108.1) | 97.0% | < 0.01 |
|  | Non-referral | 5 | 54.6 (28.4, 88.5) | 98.4% | < 0.01 |
| Country | Ethiopia | 5 | 94.79 (68.06, 125.38) | 95.5% | < 0.01 |
|  | Tanzania | 4 | 40.19 (30.26, 51.45) | 86.7% | < 0.01 |
|  | Uganda | 1 | 57.94 (52.18, 63.97) | - | < 0.01 |

**Supplementary Appendix 9: Meta-regression analysis**

Table S9.1: Multi-variable meta-regression analysis of population-based studies on perinatal mortality in East Africa.

| Factors | Coefficient | Std. err | P - value | 95% CI |
| --- | --- | --- | --- | --- |
| **Study design** |  |  |  |  |
| Cohort | 0.0540505 | 0.039899 | 0.176 | -0.0241502, 0.1322511 |
| Cross-sectional | 0.0627586 | 0.0491241 | 0.201 | -0.033523, 0.1590401 |
| **Study period** |  |  |  |  |
| After ENAP (>=2015) | 0.0133277 | 0.0261503 | 0.610 | -0.037926, 0.0645813 |
| **Risk of bias** |  |  |  |  |
| Low | 0.0532581 | 0.0650269 | 0.413 | -0.0741922, 0.1807084 |
| Moderate | 0.0932996 | 0.0633794 | 0.141 | -0.0309218, 0.217521 |
| **Residence** |  |  |  |  |
| Both urban and rural | 0.148557 | 0.0687681 | 0.031 | 0.013774, 0.28334 |
| Rural | 0.1122472 | 0.0607254 | 0.065 | -0.0.0067725, 0.2312668 |

By assuming a heterogeneity of 90%, a sensitivity analysis was done on the meta-regression, and the model revealed that study design (cohort: β = 0.05, p = 0.045; cross-sectional: β = 0.07, p = 0.036), risk of bias (moderate: β = 0.09, p = 0.043), and residence type (both urban and rural: β = 0.14, p = 0.002; rural: β = 0.11, p = 0.007) were identified as significant moderators affecting perinatal mortality rates.

Table S9.2: Sensitivity analysis (by artificially decreasing heterogeneity to 90%) on multi-variable meta-regression analysis of population-based studies on perinatal mortality in East Africa.

| Factors | Coefficient | Std. err | P - value | 95% CI |
| --- | --- | --- | --- | --- |
| **Study design** |  |  |  |  |
| Cohort | 0.053549 | 0.0266833 | 0.045 | 0.0012508, 0.1058472 |
| Cross-sectional | 0.0656461 | 0.0313609 | 0.036 | 0.0041798, 0.1271125 |
| **Study period** |  |  |  |  |
| After ENAP (>=2015) | 0.0107753 | 0.0167448 | 0.520 | -0.0220439, 0.0435945 |
| **Risk of bias** |  |  |  |  |
| Low | 0.0502675 | 0.0450696 | 0.265 | -0.0380673, 0.1386024 |
| Moderate | 0.0902876 | 0.044593 | 0.043 | 0.0028869, 0.1776883 |
| **Residence** |  |  |  |  |
| Both urban and rural | 0.1434743 | 0.0467644 | 0.002 | 0.0518178, 0.2351308 |
| Rural | 0 .1092351 | 0.0407328 | 0.007 | 0.0294003, 0.18907 |

**Supplementary Appendix 10: Sensitivity analysis**

Figure S10.1: Sensitivity analysis of population-based studies on perinatal mortality in East Africa


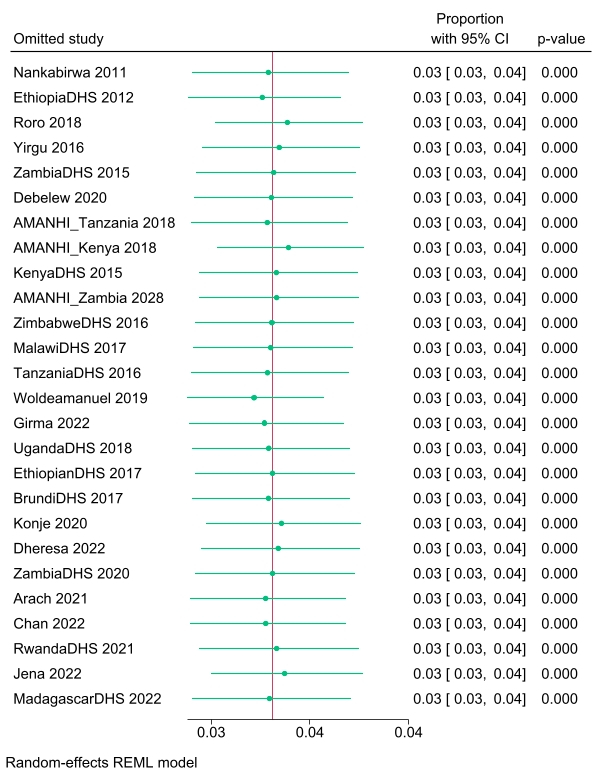


**Supplementary Appendix 11: Publication bias**

Figure S11.1: Funnel plot of population-based perinatal mortality rate in East Africa.


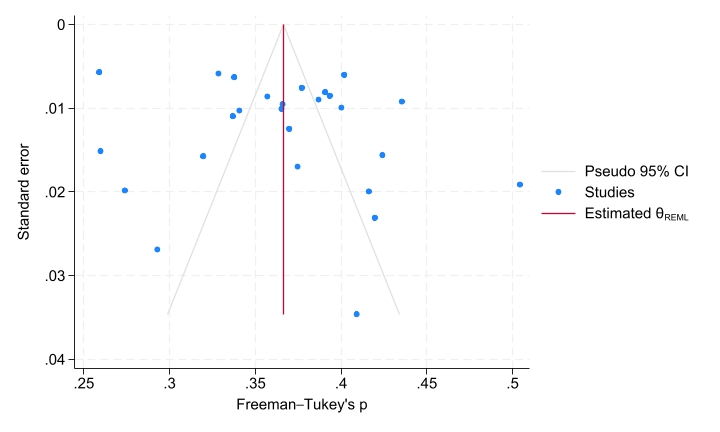


Figure S11.2: Trim and fill funnel plot of population-based perinatal mortality rate in East Africa


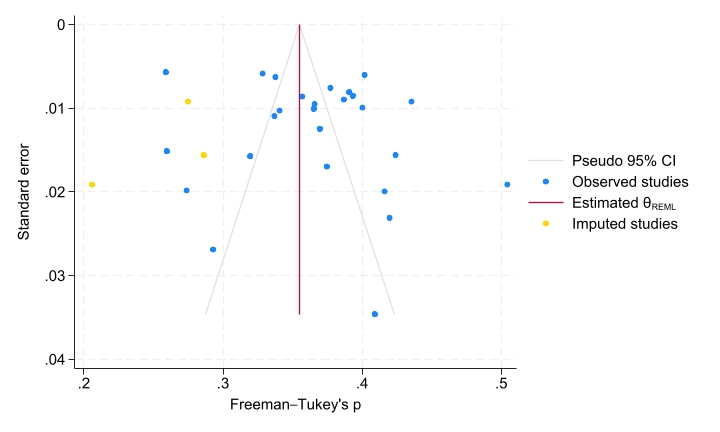


**Supplementary Appendix 12: Characteristics of studies on causes of perinatal mortality in East Africa.**

| **Author** | **Country** | **Study design** | **Classification system used** | **Data source** | **Cause of death**  **determined by** | **Study population** | **Number of deaths** |
| --- | --- | --- | --- | --- | --- | --- | --- |
| Mmbaga 2012 | Tanzania | Cross-sectional | Neonatal and Intrauterine Death Classification according to Etiology (NICE) | Medical records | Study authors | Perinatal | 1958 |
| Nankabirwa 2011 | Uganda | Cohort | No classification system was reported | Verbal Autopsy (VA) | Study authors | Perinatal | 34 |
| Nakibuuka 2012 | Uganda | Case series | No classification system was reported | Medical records | Physician | Perinatal | 120 |
| Turnbull 2011 | Zambia | Cross-sectional | No classification system | VA | Physician | Stillbirth | 50 |
| Schmiegelow 2012 | Tanzania | Cohort study | Wigglesworth | Interview and medical records | Physician | Perinatal | 46 |
| Demise 2015 | Ethiopia | Cross-sectional | Wigglesworth | VA | Physician (Audit team) | Stillbirth | 61 |
| Assefa 2016 | Ethiopia | Cohort study | ICD-10 | VA | Physician | Early Neonatal death | 248 |
| Yirgu 2016 | Ethiopia | Case-control | ICD-10 | VA | Physician | Stillbirth | 57 |
| Musafili 2017 | Rwanda | Cross-sectional | No classification system | Medical records | Physician | Perinatal | 250 |
| Roro 2018 | Ethiopia | Case-control | ICD-10 | VA | Physician | Stillbirth | 24 |
| Mengesha 2017 | Ethiopia | Cohort | Wigglesworth | VA | Physician | Early neonatal death | 51 |
| AMANHI 2018 | Kenya, Tanzania, and Zambia | Cohort | ICD-10 | VA | Physician | Stillbirth | 615 |
| Ngwenya 2018 | Zimbabwe | Cross-sectional | No classification system | Medical records | Physician/midwife | Stillbirth | 268 |
| Aminu 2019 | Kenya, Malawi, and Zimbabwe | Case series | ICD-PM | Medical records | Trained healthcare workers | Stillbirth | 1060, including cases from Sierra Leone |
| Kebede 2021 | Ethiopia | Case-control | Wigglesworth | VA and Fetal autopsy if COD unknown | Senior midwife | Stillbirth | 366 |
| Mangu 2021 | Tanzania | Cross-sectional | ICD-10 and CODAC | Medical records | Physician | Early neonatal death | 23590 |
| Alsammani 2021 | Sudan | Cross-sectional | No classification system | Structured questionnaire | Physician | Stillbirth | 285 |
| Housseine 2021 | Tanzania | Cross-sectional | ICD-PM | Medical records | Physician | Perinatal | 413 |
| Arach 2021 | Uganda | Cohort study | ICD-PM | VA | Physician | Perinatal | 37 |
| Beyene 2022 | Ethiopia | Cross-sectional | No classification system | Medical records | Not clear (from medical record) | Perinatal | 1129 |
| Tesfay 2022 | Ethiopia | Cross-sectional | ICD-PM | Medical records | MPDSR committee | Stillbirth | 1417 |

**Supplementary Appendix 13: Updated conceptual framework for causes and risk factors of perinatal mortality.**

**Socio-demographic, behavioural and lifestyle factors**

- Income (household wealth index)
- Education
- Occupation
- Drinking alcohol
- Environmental risk factors
- Place of residence
- Intimate partner violence
- Media exposure
- Family size
- Marital status
- Female-headed household
- Women’s participation in healthcare decision

**Health facility-related factors**

- Place of delivery
- Unintended pregnancy
- Healthcare financing
- Geographic risk (distance to health facility)
- Referral system

**Distal Determinants**

**Obstetrics risk factors**

- Mother’s age
- Multiple birth
- Mode of delivery
- Maternal medical and surgical conditions
- Malnutrition (over/under)
- Number and quality of antenatal care
- Previous history of adverse pregnancy outcome
- STI including HIV/AIDS

**Quality of care**

- Delivery assisted by trained health workers
- Labour induction/ augmentation
- Use of partograph
- Maternity waiting homes

**Proximate Determinants**

**Fetal factors**

- Sex
- Gestational age
- Birth weight
- Length of birth interval
- Seasonal risk factor
- Small for gestational age
- APGAR score

**Obstetric complications**

- Antepartum haemorrhage (Abruptio placenta, placenta previa)
- Hypertensive disorder of pregnancy
- Obstructed labour
- Prolonged labour
- Premature Rupture of Membrane (PROM)
- Maternal infection

**Underlying Causes of Death**

- Prematurity
- Growth Restriction
- Intrauterine Hypoxia
- Congenital Anomaly

**Immediate Causes of Death**

- Perinatal Asphyxia
- Trauma
- Infection/Sepsis

**Cause of death**

**Perinatal Mortality**

**Supplementary Appendix 14: Forest plots of risk factors of perinatal mortality in East Africa.**

Figure S14.1: Socio-demographic, behavioural and lifestyle factors


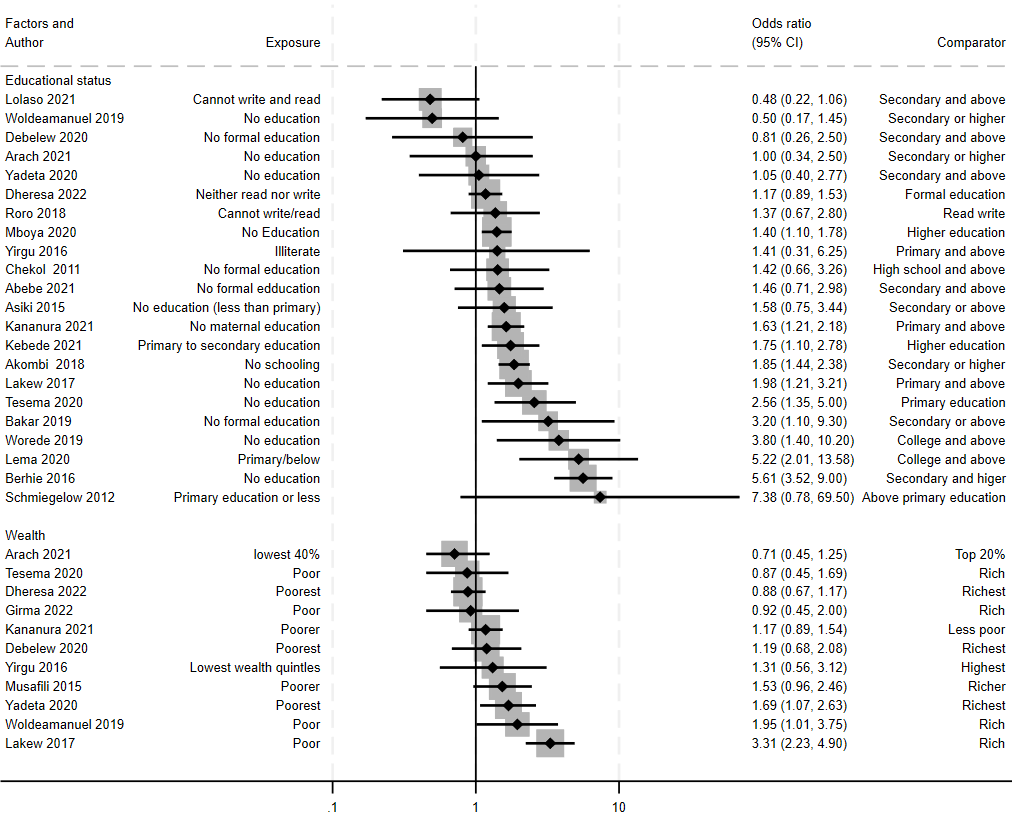


**Continued**

**
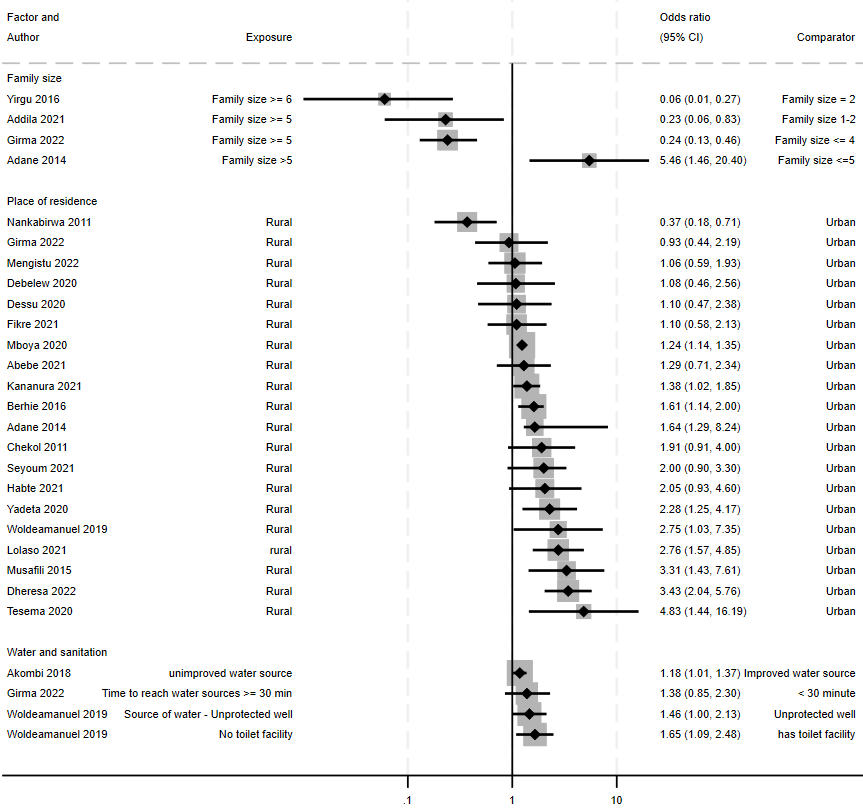
**

**Continued**

**
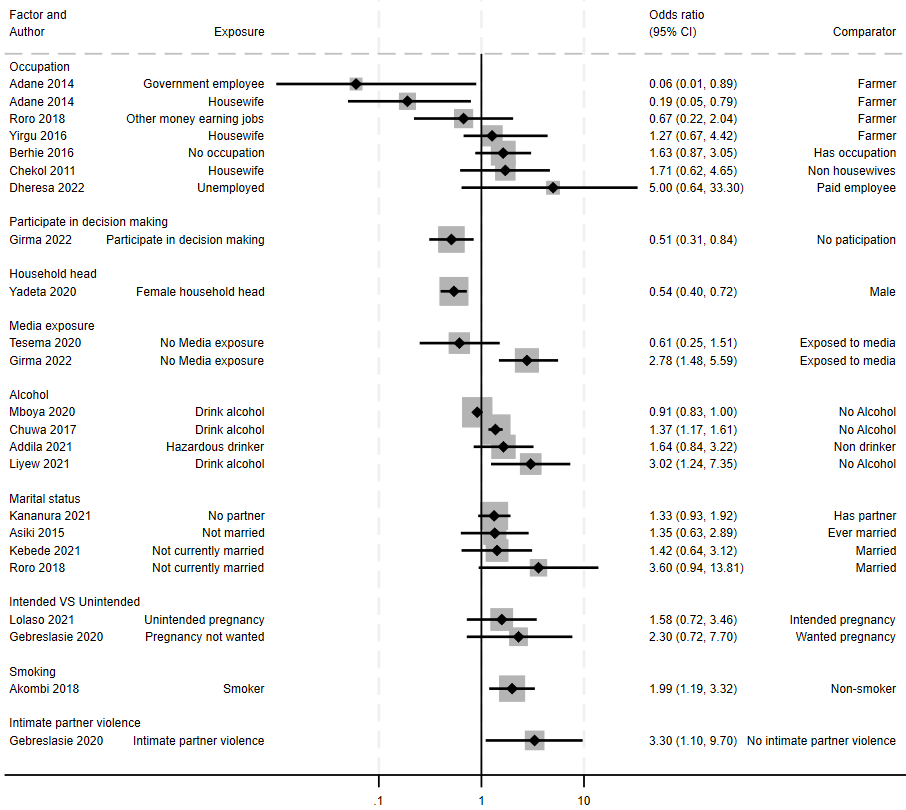
**

Figure S14.2: Obstetric risk factors


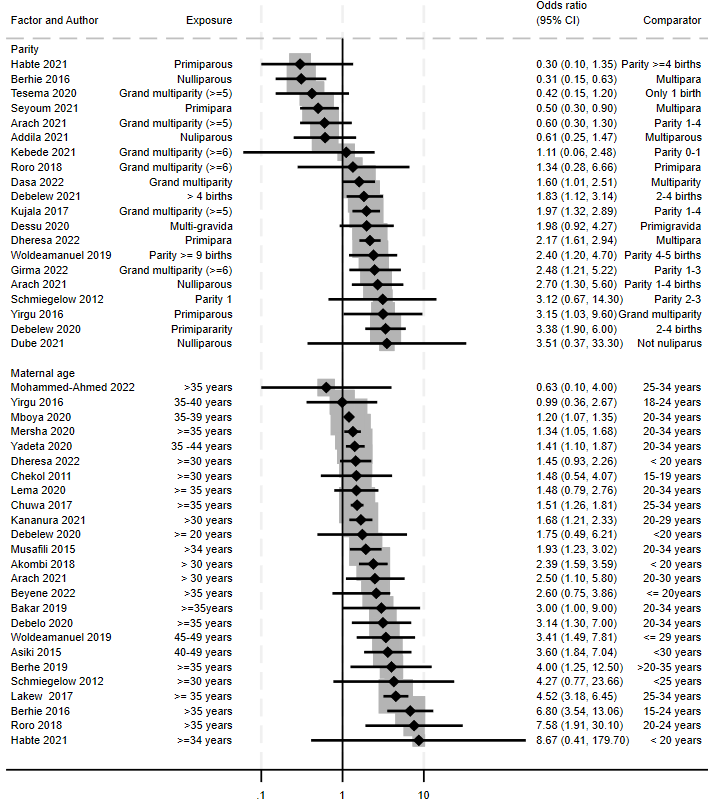


Continued


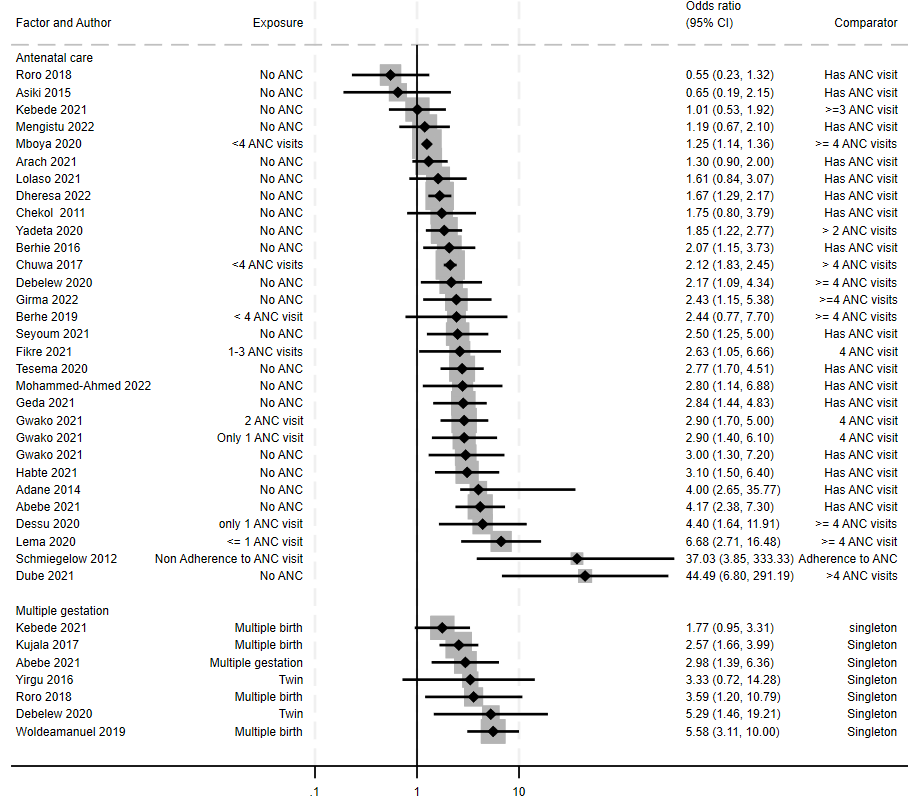


Continued


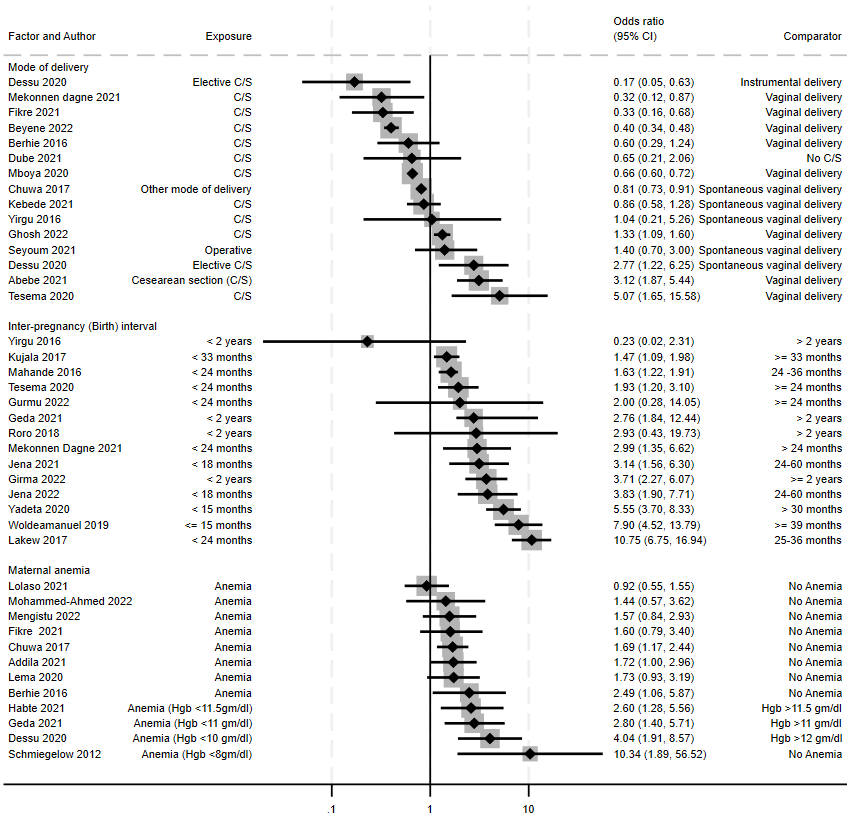


Continued


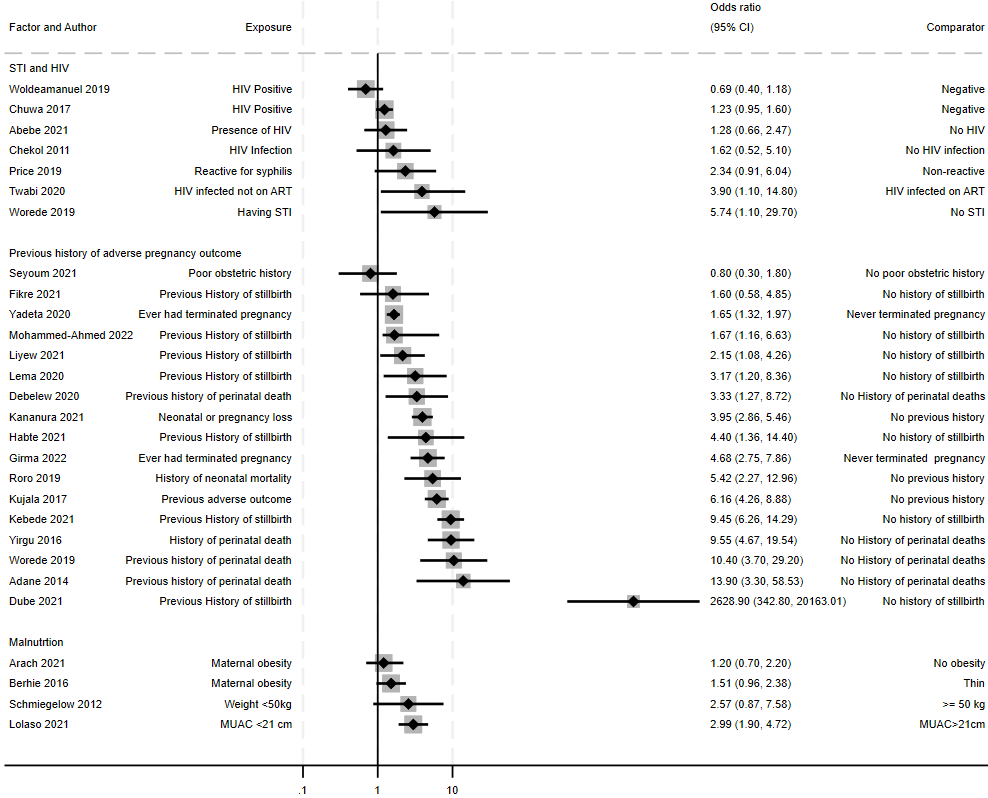


Figure S14.3: Obstetric complications


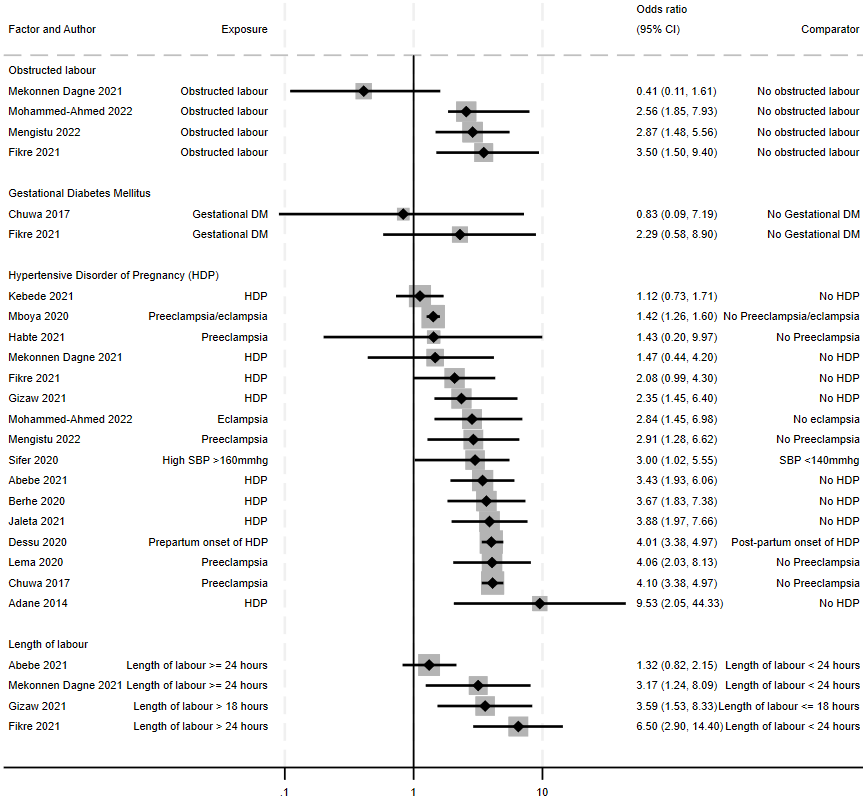


Continued


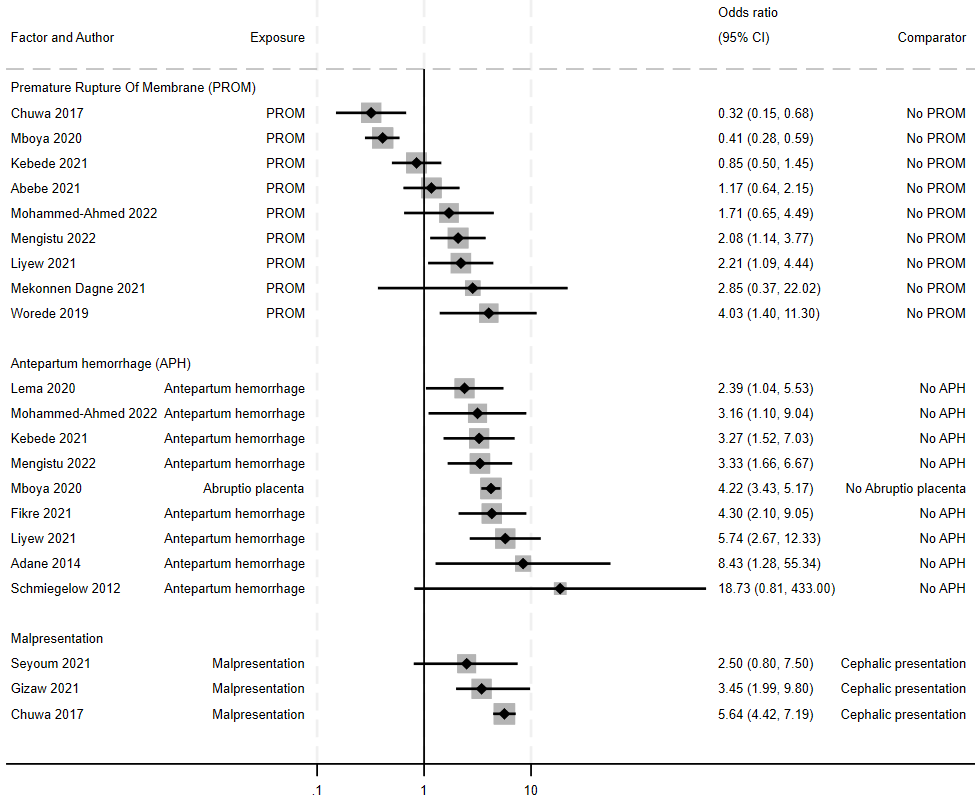


Figure S14.4: Fetal Factors


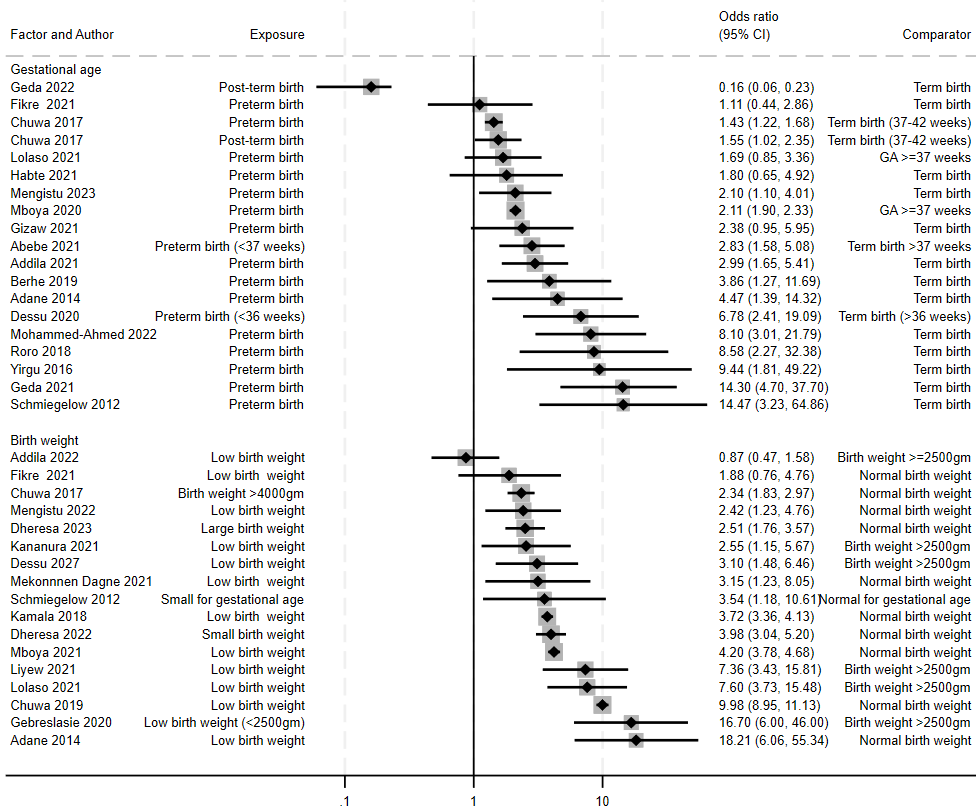


Continued


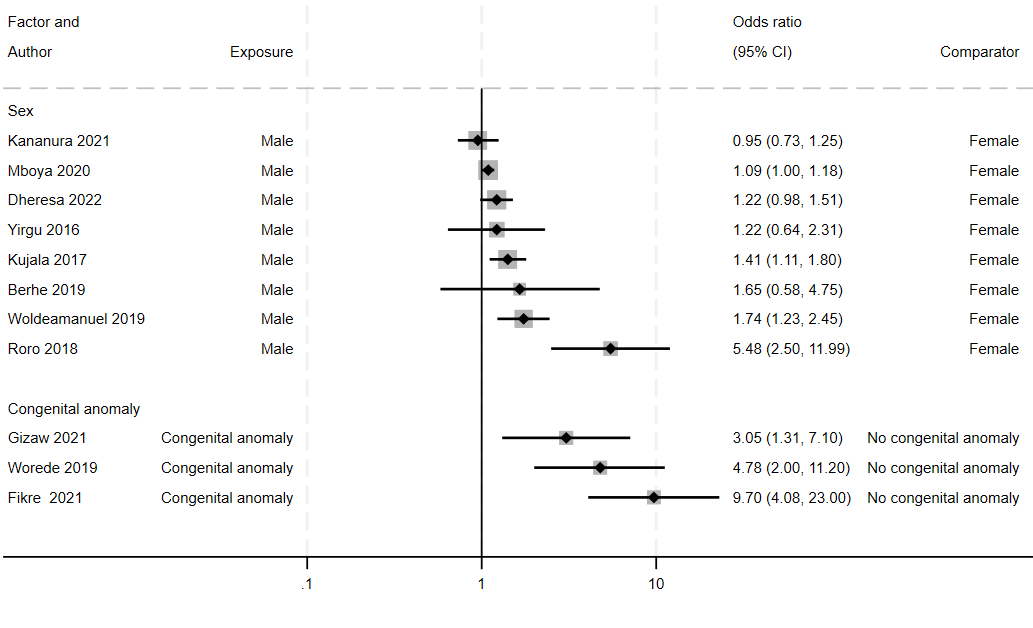


Figure S14.5: Health system factors


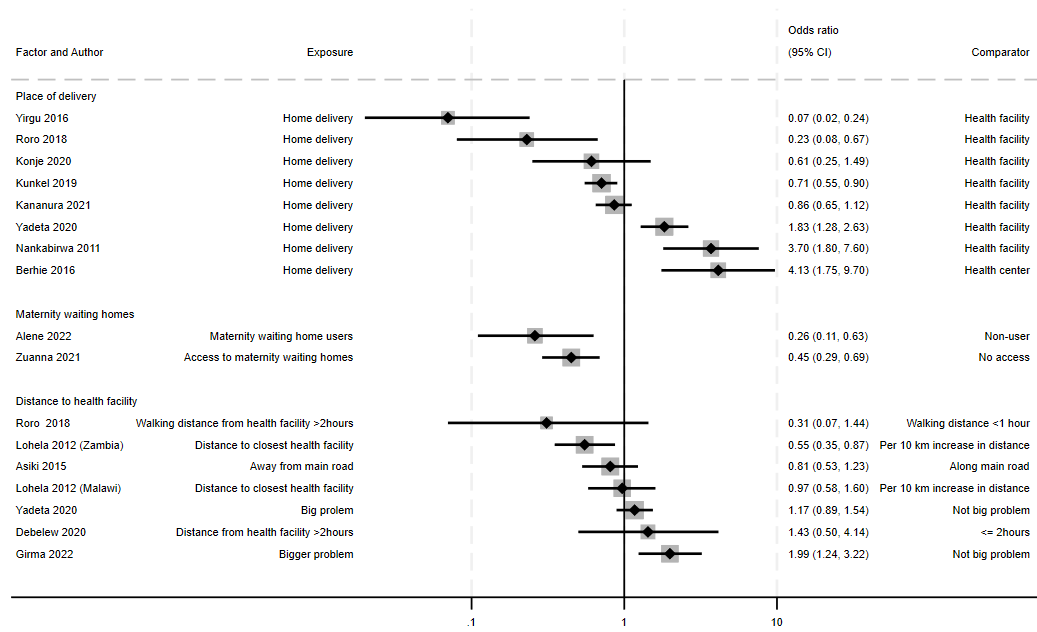


Continued


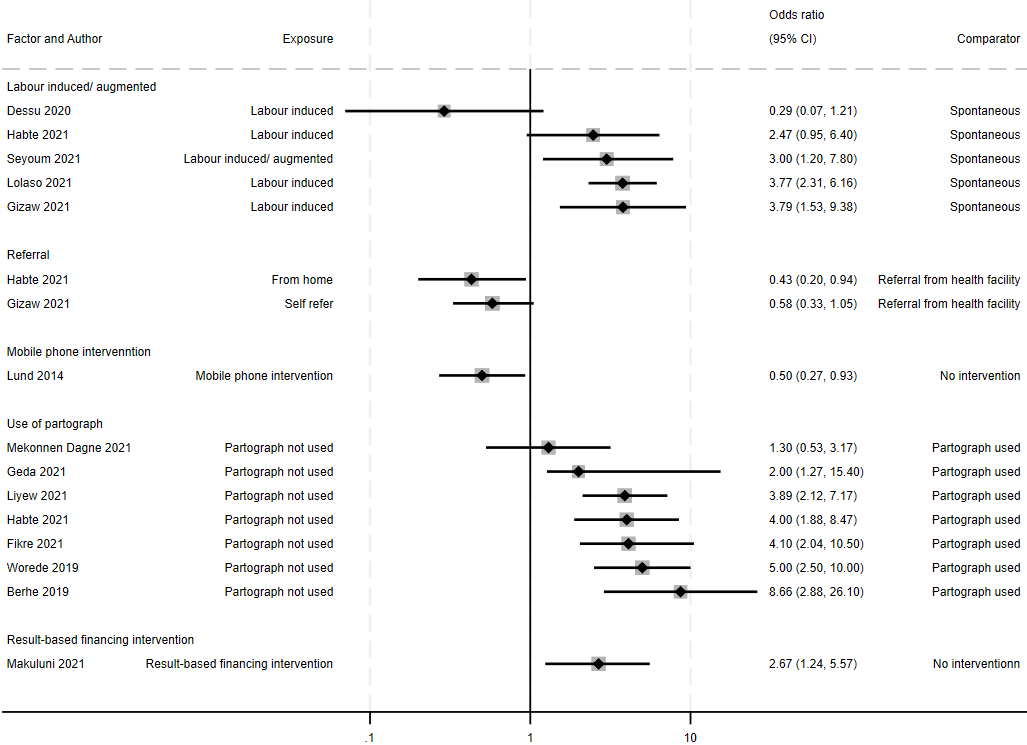

Supplement: Supplementary file 1 — Appendix S1. [file IJGO-173-1207-s001.docx]
